# Supplementary material for: Push-Pull Effect of Terpyridine Substituted by Triphenylamine Motive—Impact of Viscosity, Polarity and Protonation on Molecular Optical Properties
Source: Molecules. 2022 Oct 20;27(20):7071. doi: 10.3390/molecules27207071 (PMC9606908; doi:10.3390/molecules27207071)
Supplement: Supplementary file 1 [file molecules-27-07071-s001.zip › molecules-1968107-supplementary.pdf]

SUPPORTING INFORMATION
Push-pull effect of terpyridine substituted by triphenylamine motive – impact of viscosity, polarity and protonation on molecular optical properties

Anna Maria Maroń[1,*], Oliviero Cannelli[2,*], Etienne Christophe Socie[3], Piotr Lodowski[1], Barbara Machura[1]

[1] Institute of Chemistry, University of Silesia, 9th Szkolna Str., 40-006 Katowice, Poland. [2] Laboratory of Ultrafast Spectroscopy (LSU) and Lausanne Centre for Ultrafast Science (LACUS), École Polytechnique Fédérale de Lausanne, CH-1015, Lausanne, Switzerland.

[3] Photochemical Dynamics Group, Institute of Chemical Sciences and Engineering (ISIC), École Polytechnique Fédérale de Lausanne, CH-1015, Lausanne, Switzerland.

* email: anna.maron@us.edu.pl, oliviero.cannelli@epfl.ch

Supporting Information Contents

Scheme S1. 4′-(4-(di(4-tert-butylphenyl)amine)phenyl)-2,2′,6′,2′′-terpyridine (tBuTPAterpy) and the model chromophores considered at this study 3

Synthetic procedure and identification of tBuTPAterpy from [1] 3

Figure S1. ^{1}H NMR and ^{13}C NMR spectra of tBuTPAterpy 4

Figure S2. FT-IR spectrum of tBuTPAterpy 5

Steady-state spectra 6

Table S1. Absorption and emission properties of tBuTPAterpy in comparison to its building blocks: (i) 2,2′:6′,2′′-terpyridine (terpy) and bis(4-tert-butylphenyl)aniline (tBuTPA), and (ii) 4′-phenyl-2,2′:6′,2′′-terpyridine (4′-Ph-terpy) and bis(4-tert-butylphenyl)amine (tBuDPA) in *n*-hexane solutions 6

Figure S3. Absorption (Abs), emission (PL) and excitation (PE) spectra of tBuTPAterpy in solvents of various polarity at room temperature. 12

Figure S4. Comparison of roomT (purple) and 77 K (lowT, blue) photoexcitation (PE) and photoemission (PL) spectra of tBuTPAterpy in solvent of various polarity. 17

Time correlated single photon counting measurements 17

Figure S5. PL decay curves of tBuTPAterpy in various solvents. 29

Table S2. Spectral properties of tBuTPAterpy at room temperature. 30

Table S3. Spectral properties of tBuTPAterpy at 77 K. 31

Figure S6. Solvent polarity $E_T(30)$ (kcal·mol^{-1}) dependence of: absorption band FWHM (cm^{-1}) (black), extinction coefficient of the absorption maximum (M^{-1} cm^{-1}) (red), photoluminescence quantum yield (a.u.) (green) and lifetime (ns) (blue). 32

Figure S7. (a) tBuTPAterpy emission intensity and (b) PL decay time in BuCN at room temperature (roomT, red) and 77 K (lowT, blue). 33

Time resolved emission maps 34

Figure S8. TRES maps of tBuTPAterpy in various solvents and temperatures. 36

Figure S9. (a) Normalized PL spectra as a function of the titration of a tBuTPAterpy (1×10^{-5} M) chloroform solution with trifluoroacetic acid (TFA) (1-1000 equivalents). (b) Normalized excitation (dash line) and emission (solid line) spectra of neutral (black), protonated (red) and deprotonated (blue) forms of tBuTPAterpy. 37

Figure S10. Comparison of the tBuTPAterpy decay curves for the protonated (black) and neutral (pink) form. 38

DFT calculations 39

Table S4. tBuTPAterpy five lowest singlet electronic excitation and the $S_1\rightarrow S_0$, $T_1\rightarrow S_0$ de-excitation energies. 39

Figure S11. Kohn - Sham orbitals involved in six lowest electronic excitations for tBuTPAterpy. The character of the individual orbitals and the energy order are almost independent of the type of solvent used in the PCM model. 40

Figure S12. (a) Rotation angles between the central phenyl ring and the terpyridine (θ) and amino (ϕ) substituents, respectively. (b) Potential energy curves for S_0 ground state and the two lowest excited electronic states S_1 and S_2 as function of θ and ϕ dihedral angles. (c) Dependence of the oscillator strength on the rotation angle θ and ϕ for the singlet transitions S_1 and S_2. All simulations were preformed using the ACN solvent in the PCM model. 41

References 42

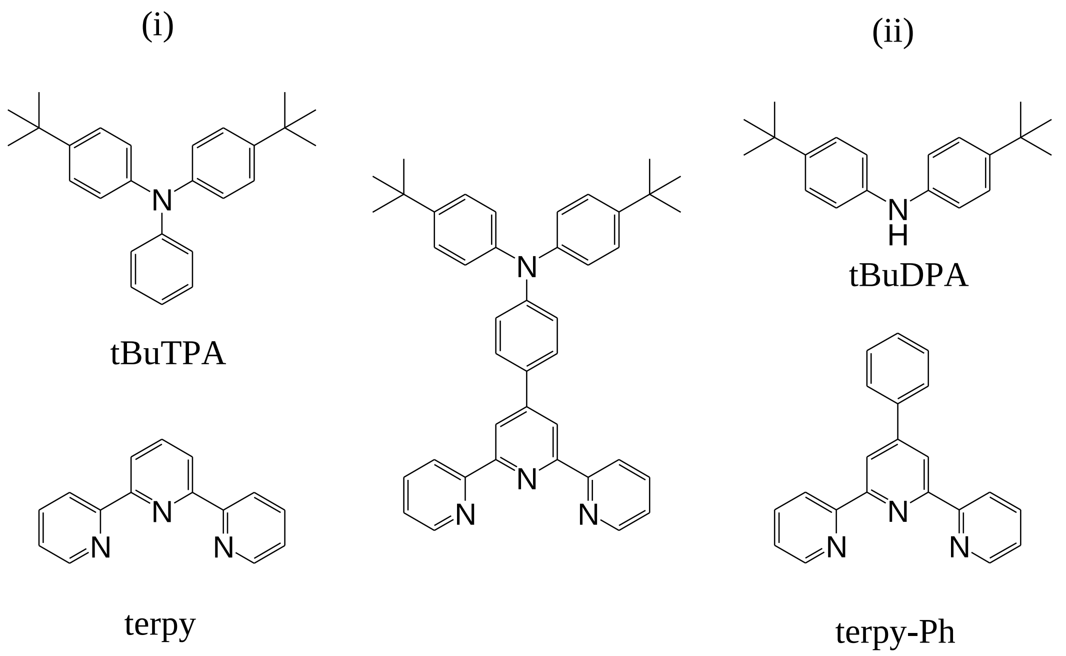

Scheme S1. 4′-(4-(di(4-tert-butylphenyl)amine)phenyl)-2,2′,6′,2″-terpyridine (**tBuTPAterpy**) and the model chromophores considered at this study

Synthetic procedure and identification of tBuTPAterpy from [1]

A mixture of 4′-(4-bromophenyl)-2,2′,6′,2″-terpyridine (1.72 g, 4.44 mmol), bis(4-tert-butylphenyl)amine (1.5 g, 5.33 mmol), $Pd(OAc)_2$ (40 mg, 0.18 mmol, 4%-mol), $P(^tBu)_3$ (73 mg, 0.36 mmol, 8%-mol) and NaO^tBu (770 mg, 8 mmol) in 100 mL of anhydrous toluene was heated under reflux for 24 h under argon atmosphere. Then, the mixture was cooled to room temperature, water (60 mL) was added, and the mixture was stirred for 20 minutes. The mixture was extracted with dichloromethane (30 mL) three times. The combined organic extract was washed with brine and dried with anhydrous Na_2SO_4. The solvent was removed by a rotary evaporator under vacuum. The crude product was purified by column chromatography (silica gel, AcOEt/hexane/$NH_3 \cdot H_2O$ 75:50:1 v/v) and recrystallized from ethanol, to give tBuTPAterpy as a yellow solid(1.64 g, 63%). **^{1}H NMR** (400 MHz, $CDCl_3$) δ 8.73 – 8.71 (m, 4H), 8.66 (d, *J* = 7.8 Hz, 2H), 7.87 (t, *J* = 7.7 Hz, 2H), 7.77 (d, *J* = 8.5 Hz, 2H), 7.36 – 7.28 (m, 6H), 7.15 (d, *J* = 8.5 Hz, 2H), 7.09 (d, *J* = 8.4 Hz, 4H), 1.34 (s, 18H). **^{13}C NMR** (100 MHz, $CDCl_3$) δ 156.54, 155.91, 149.88, 149.24, 149.19, 146.34, 144.77, 136.84, 131.03, 128.04, 126.27, 124.55, 123.76, 122.38, 121.38, 118.28, 34.45, 31.57. $C_{41}H_{40}N_4$ (588.79 g/mol) calcd: C, 83.64; H, 6.85; N, 9.52%. Found: C, 83.64; H, 6.95; N, 9.58%.

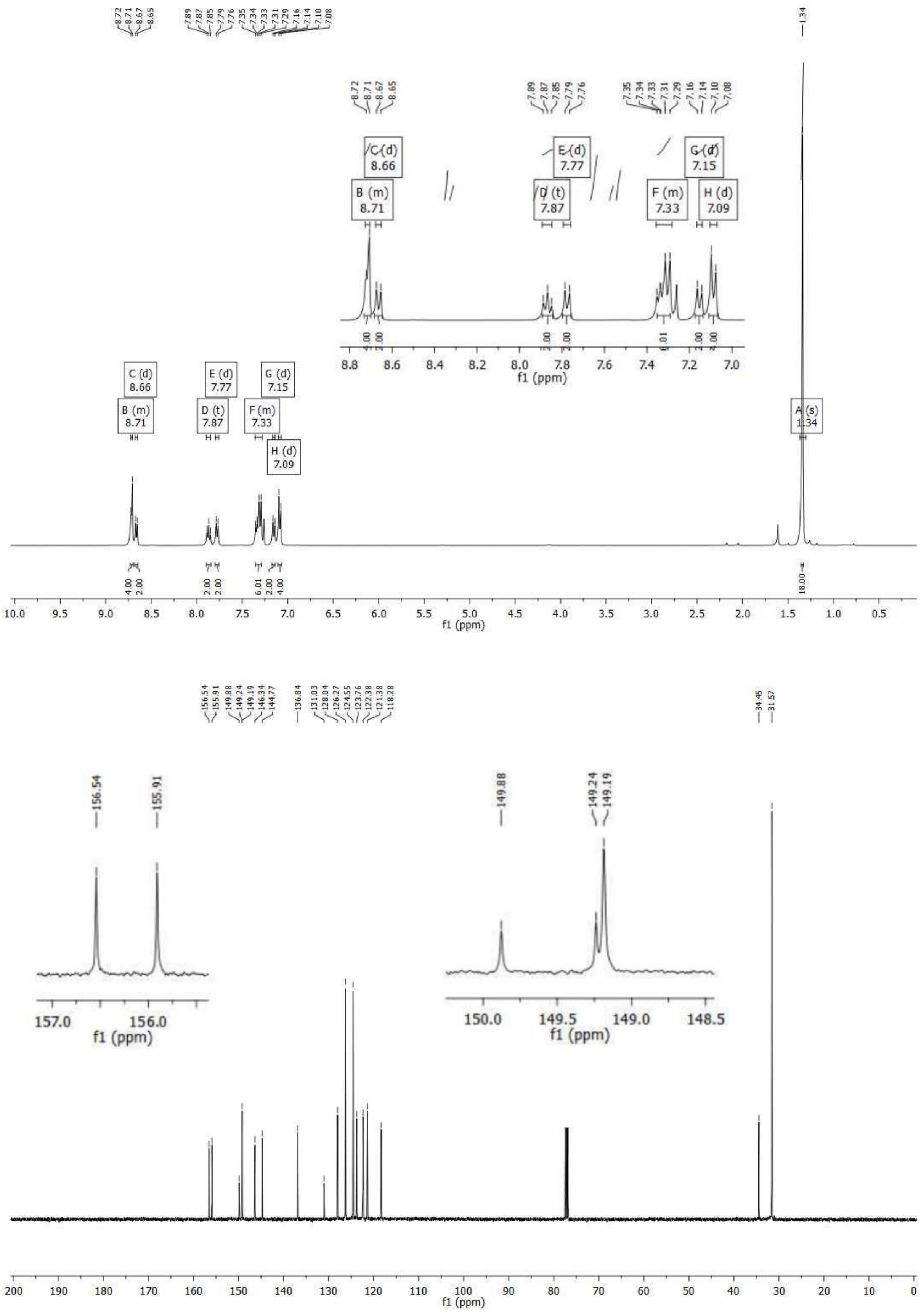

Figure S1. ^{1}H NMR and ^{13}C NMR spectra of **tBuTPAterpy**

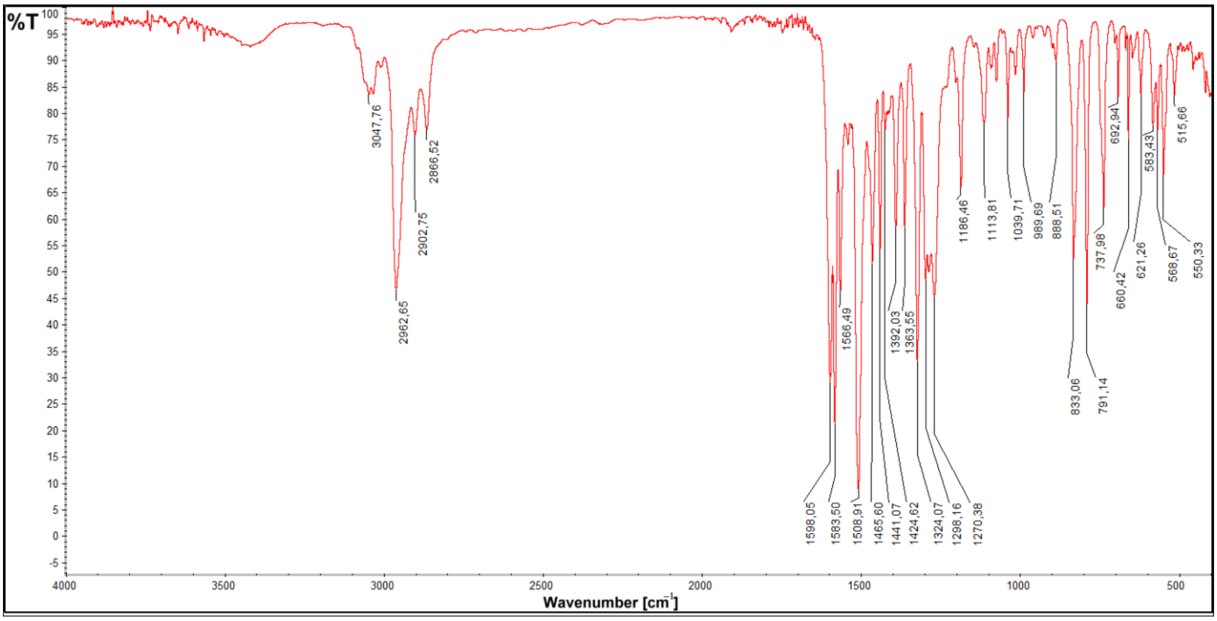

Figure S2. FT-IR spectrum of **tBuTPAterpy**

Steady-state spectra

UV-Visible measurements were carried out in solutions using an Evolution 220 (ThermoScientific) spectrophotometer (Figure S3). Steady-state photoluminescence emission spectra were recorded with FLS-980 fluorescence spectrophotometer (Edinburgh Instruments) in solutions at room temperature and at 77 K (Figures S3-S4). A 450 W Xe lamp and a photomultiplier (PMT) (Hamamatsu, R928P, Japan) detector were used as light source and detector, respectively. An emission correction file was applied to take into account for the sensitivity of the monochromator, detector, sphere coating and optics to different wavelengths. The excitation wavelength was set to 375 nm for the measurements of emission spectra. For the obtained emission maxima, excitation spectra were collected. Both the emission and excitation scans were performed with 1 nm step, 0.2 dwell time and repeated 2 times. The quantum yields were determined using the integrating sphere absolute method. In each measurement, a pure solvent was used as reference. Each scan was measured with 0.25 nm step, 0.2 dwell time and repeated 3 times. The FLS-980 software was used to determine the quantum yield values. Temperature-dependent emission spectra were recorded in MeOH:EtOH solution (c = 10μM) using a liquid nitrogen cryostat (Optistat DN, Oxford Instruments) equipped with a Mercury iTC temperature controller (Oxford Instrument).

Table S1. Absorption and emission properties of tBuTPAterpy in comparison to its building blocks: (i) 2,2´:6´,2´´-terpyridine (terpy) and bis(4-tert-butylphenyl)aniline (tBuTPA), and (ii) 4´-phenyl-2,2´:6´,2´´-terpyridine (4´-Ph-terpy) and bis(4-tert-butylphenyl)amine (tBuDPA) in *n*-hexane solutions

	Compound	λ_{abs}, nm (ε, $10^3 \cdot [M^{-1}cm^{-1}]$)	λ_{PL}, nm
	**tBuTPAterpy**	364 (87.4), 291 (107.4)	407
(i):	**tBuTPA**	301 (75.8)	359
	**terpy**	311 (21.4) (sh), 302 (31.0), 277 (44.8)	332
(ii):	**tBuDPA**	284 (54.7)	336
	**Ph-terpy**	310 (22.0), 274 (77.8), 252 (95.9)	337, 349(sh)

(**Abbreviations:** nHex – *n*-hexane, CHx – cyclohexane, Tol – toluene, $CHCl_3$ – chloroform, EtAc – ethyl acetate, THF - tetrahydrofuran, DCM – dichloromethane, DMF – dimethylformamide, DMSO – dimethylsulfoxide, ACN – acetonitrile, MeOH – methanol)

nHex

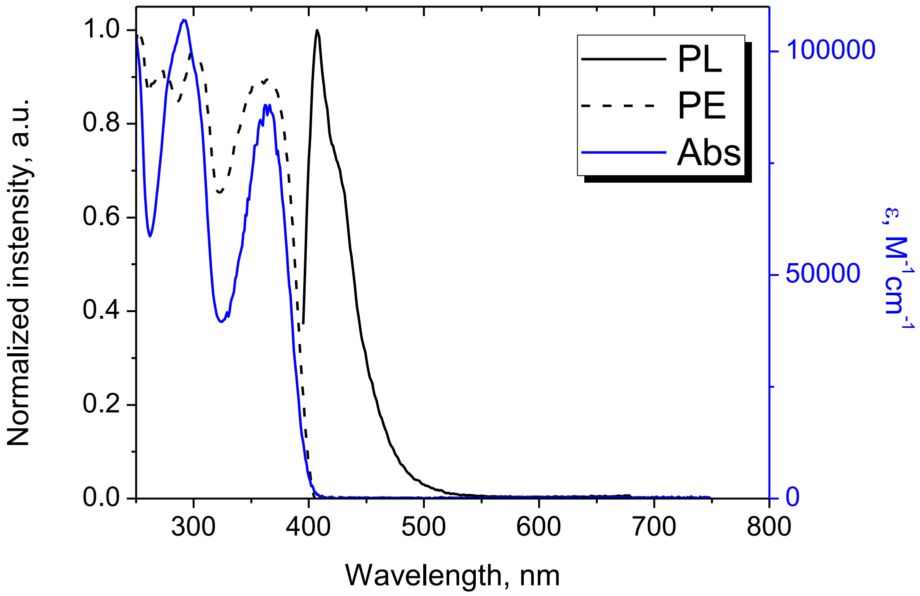

CHx

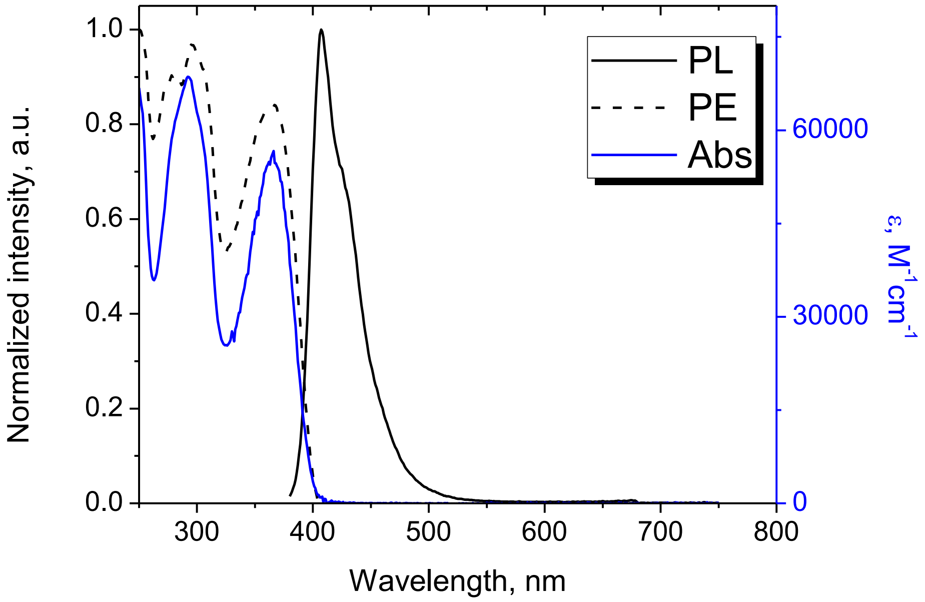

Tol

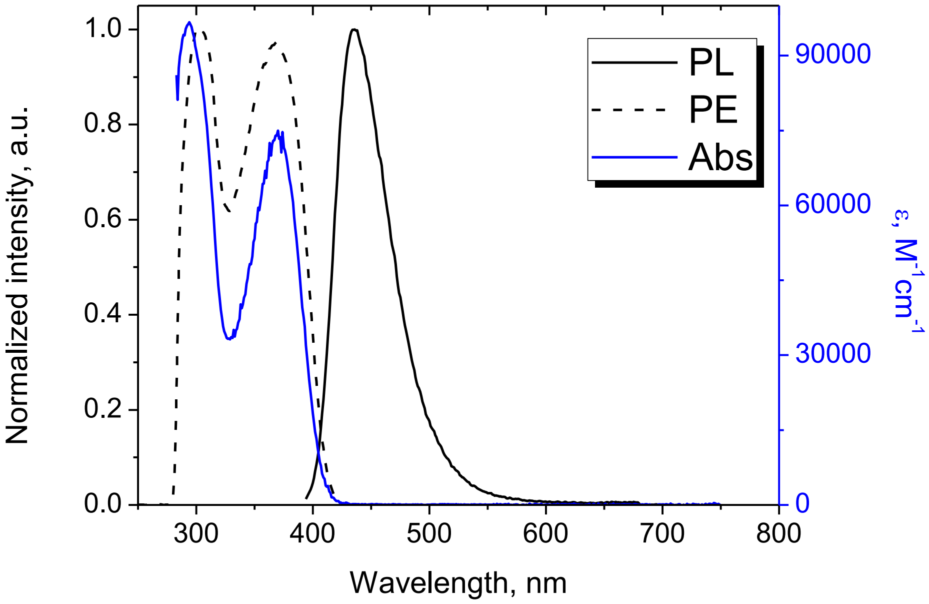
PL
PE
Abs
Normalized intensity, a.u.
Wavelength, nm
ε, $M^{-1}cm^{-1}$
1.0
0.8
0.6
0.4
0.2
0.0
300
400
500
600
700
800
90000
60000
30000
0

$CHCl_3$[1]

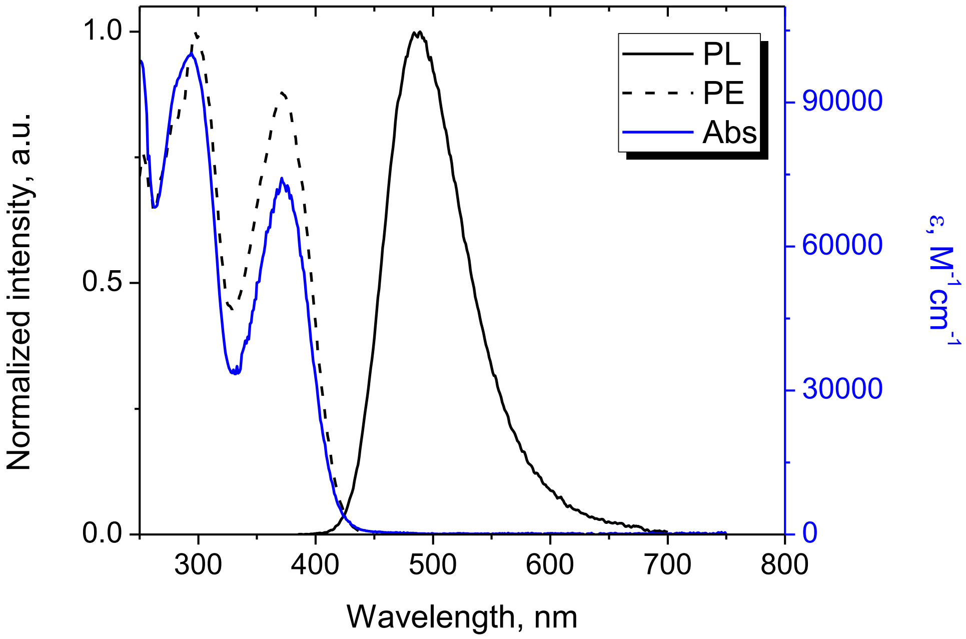
PL
PE
Abs
Normalized intensity, a.u.
Wavelength, nm
ε, $M^{-1}cm^{-1}$
1.0
0.5
0.0
300
400
500
600
700
800
90000
60000
30000
0

EtAc
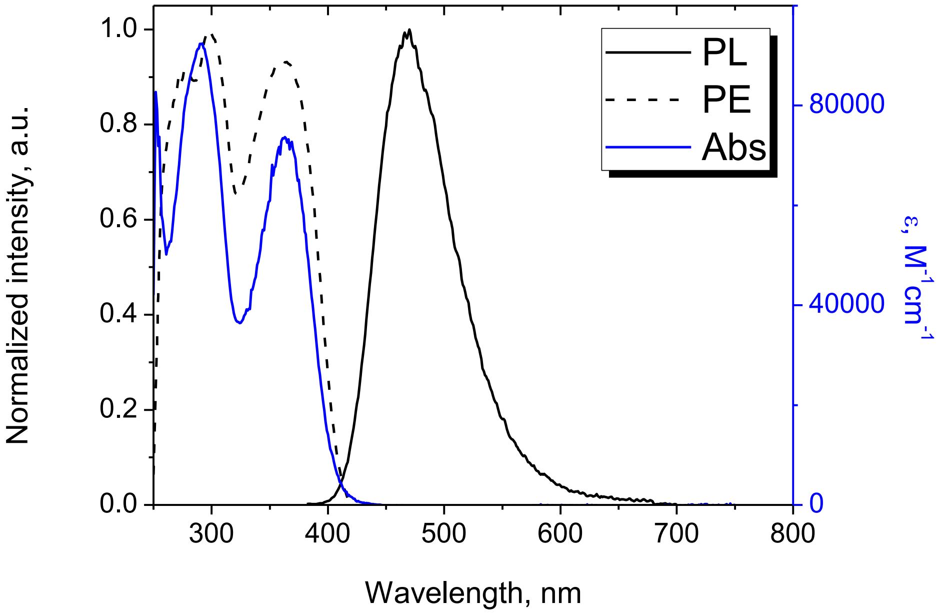PL<br>PE<br>Abs<br>Normalized intensity, a.u.<br>ε, M-1cm-1<br>Wavelength, nm<br>0.0 0.2 0.4 0.6 0.8 1.0<br>0 40000 80000<br>300 400 500 600 700 800
**THF**
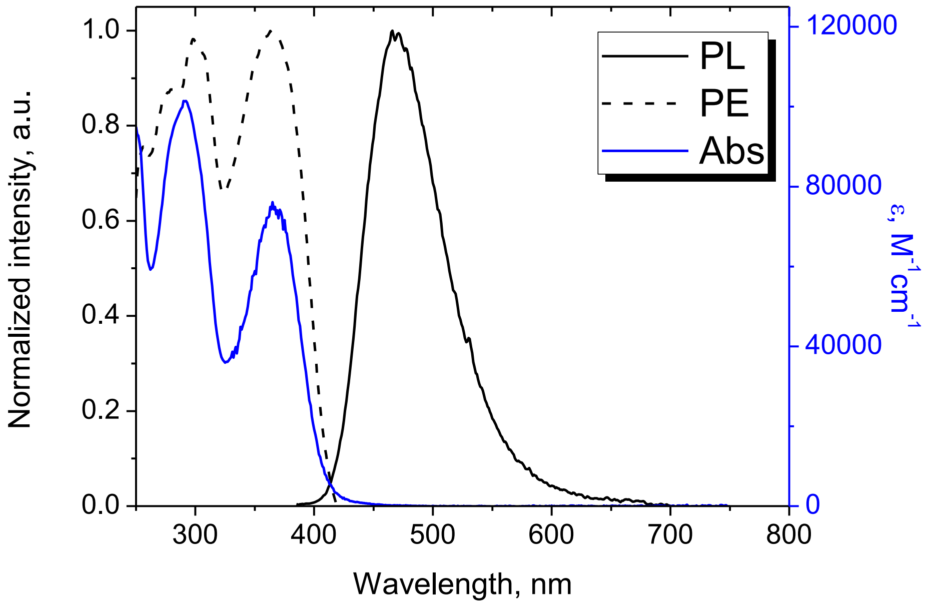PL<br>PE<br>Abs<br>Normalized intensity, a.u.<br>ε, M-1cm-1<br>Wavelength, nm<br>0.0 0.2 0.4 0.6 0.8 1.0<br>0 40000 80000 120000<br>300 400 500 600 700 800

DCM

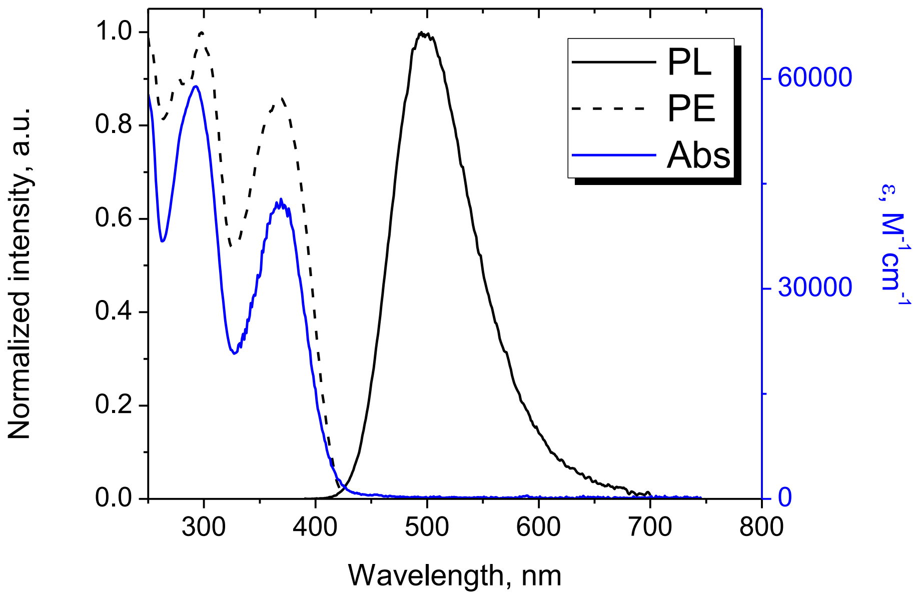
PL
PE
Abs
Normalized intensity, a.u.
Wavelength, nm
ε, M^-1 cm^-1
0.0
0.2
0.4
0.6
0.8
1.0
300
400
500
600
700
800
0
30000
60000

DMF

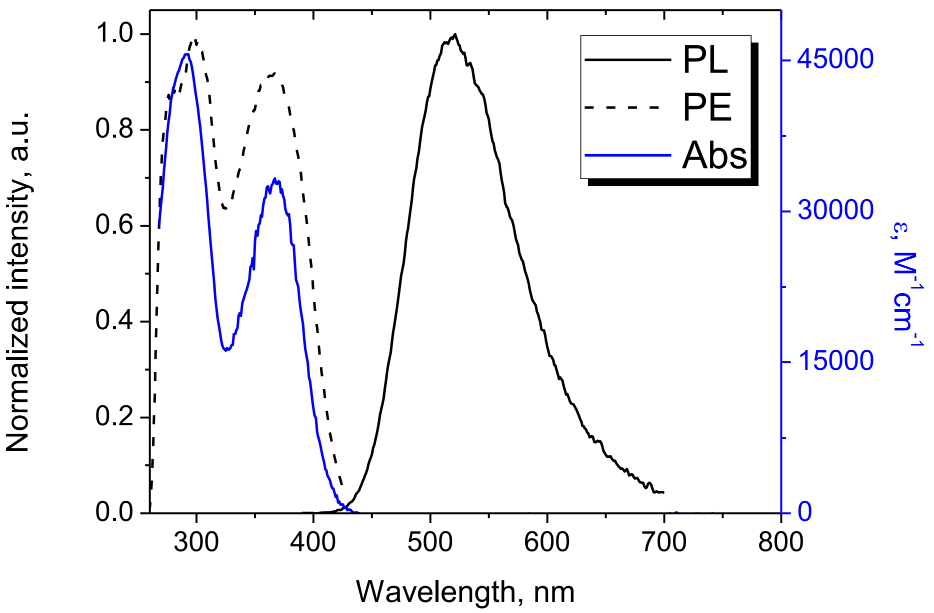
PL
PE
Abs
Normalized intensity, a.u.
Wavelength, nm
ε, M^-1 cm^-1
0.0
0.2
0.4
0.6
0.8
1.0
300
400
500
600
700
800
0
15000
30000
45000

DMSO
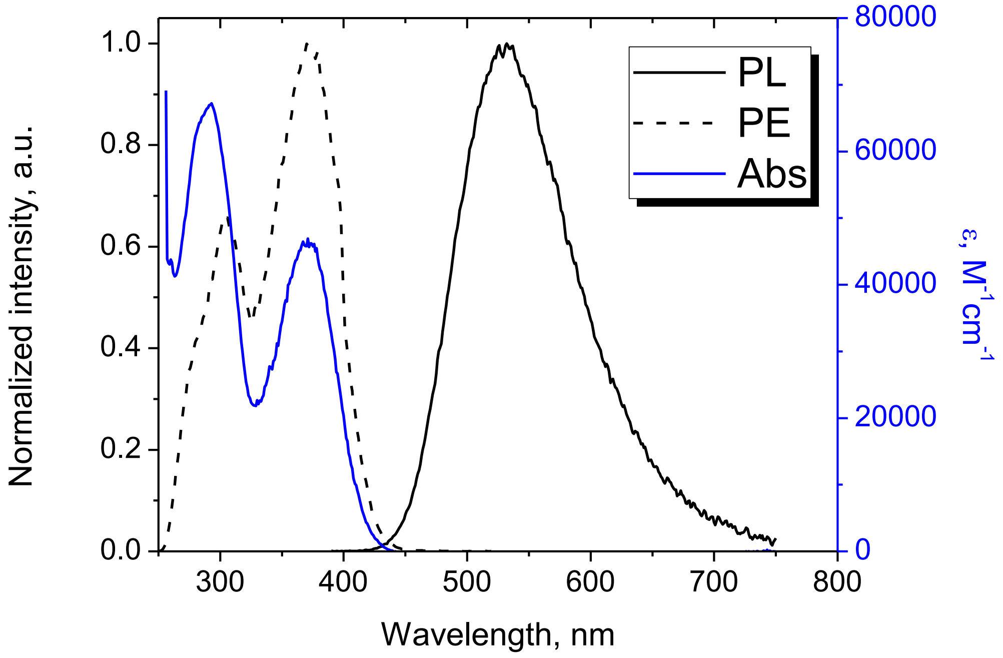Normalized intensity, a.u.<br>Wavelength, nm<br>ε, M-1 cm-1<br>PL<br>PE<br>Abs
**ACN** [1]
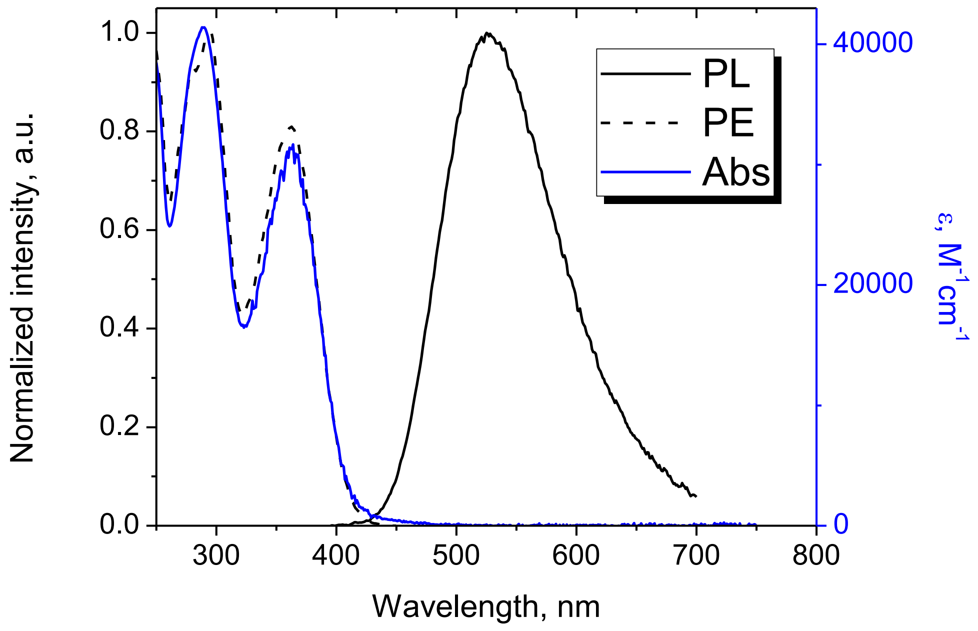Normalized intensity, a.u.<br>Wavelength, nm<br>ε, M-1 cm-1<br>PL<br>PE<br>Abs

MeOH
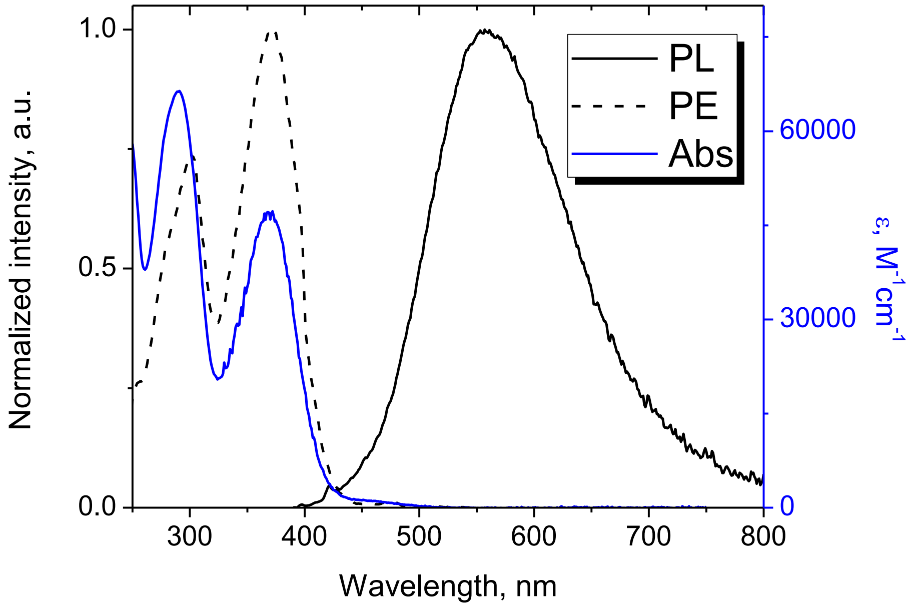

Figure S3. Absorption (Abs), emission (PL) and excitation (PE) spectra of **tBuTPAterpy** in solvents of various polarity at room temperature.

nHex

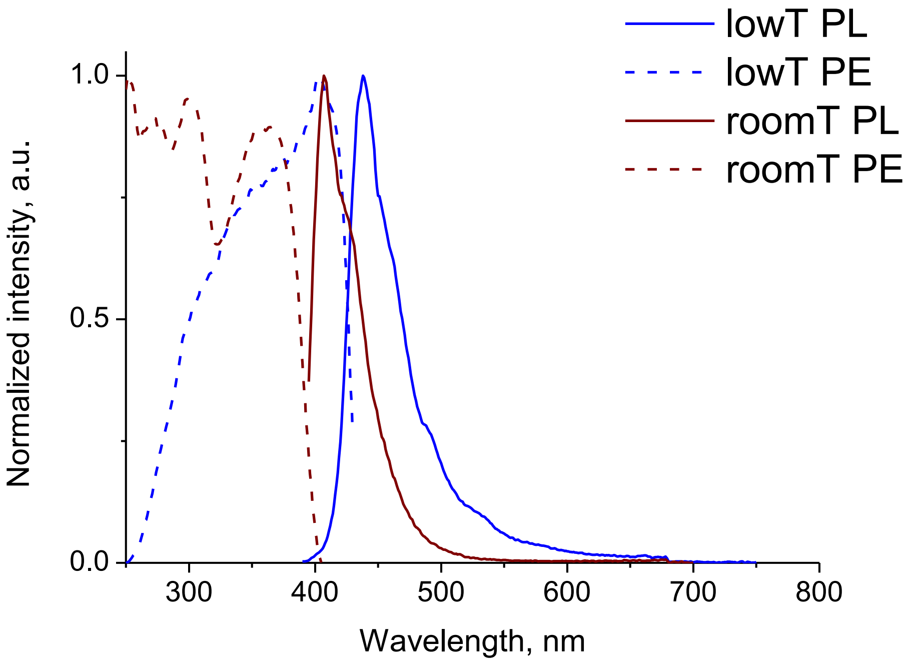
lowT PL
lowT PE
roomT PL
roomT PE
Normalized intensity, a.u.
0.0
0.5
1.0
300
400
500
600
700
800
Wavelength, nm

CHx

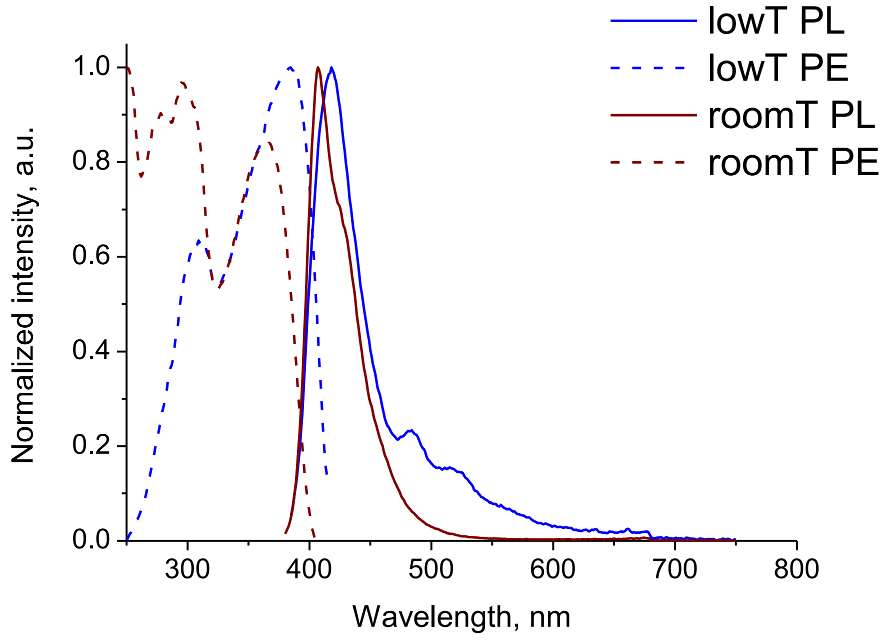
lowT PL
lowT PE
roomT PL
roomT PE
Normalized intensity, a.u.
0.0
0.2
0.4
0.6
0.8
1.0
300
400
500
600
700
800
Wavelength, nm

Tol

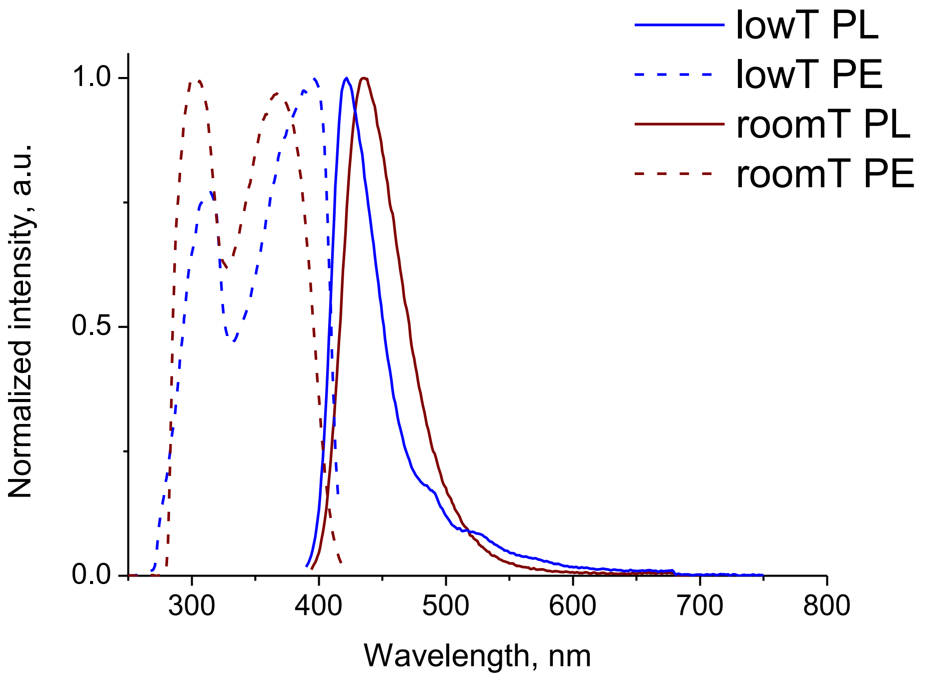
lowT PL
lowT PE
roomT PL
roomT PE
Normalized intensity, a.u.
1.0
0.5
0.0
300
400
500
600
700
800
Wavelength, nm

EtAc

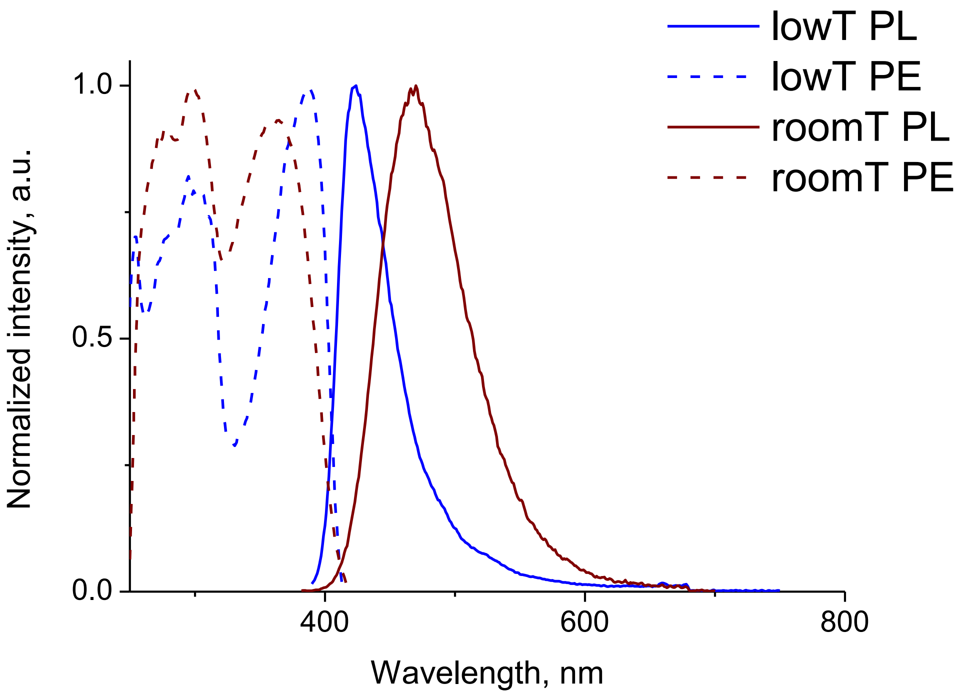
lowT PL
lowT PE
roomT PL
roomT PE
Normalized intensity, a.u.
1.0
0.5
0.0
400
600
800
Wavelength, nm

THF

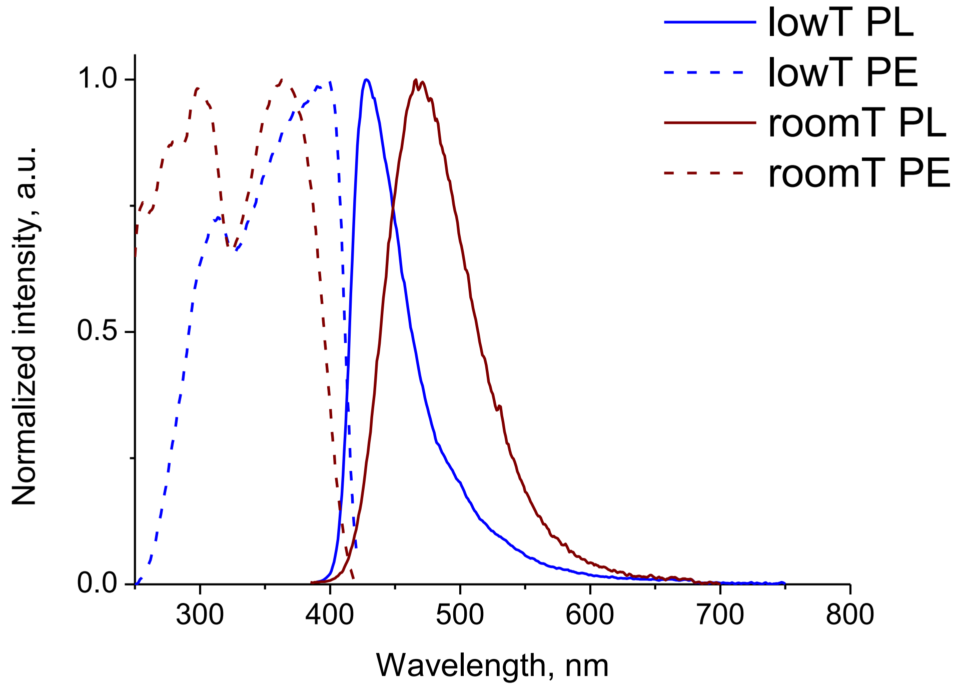

lowT PL
lowT PE
roomT PL
roomT PE
Normalized intensity, a.u.
1.0
0.5
0.0
300
400
500
600
700
800
Wavelength, nm

DCM

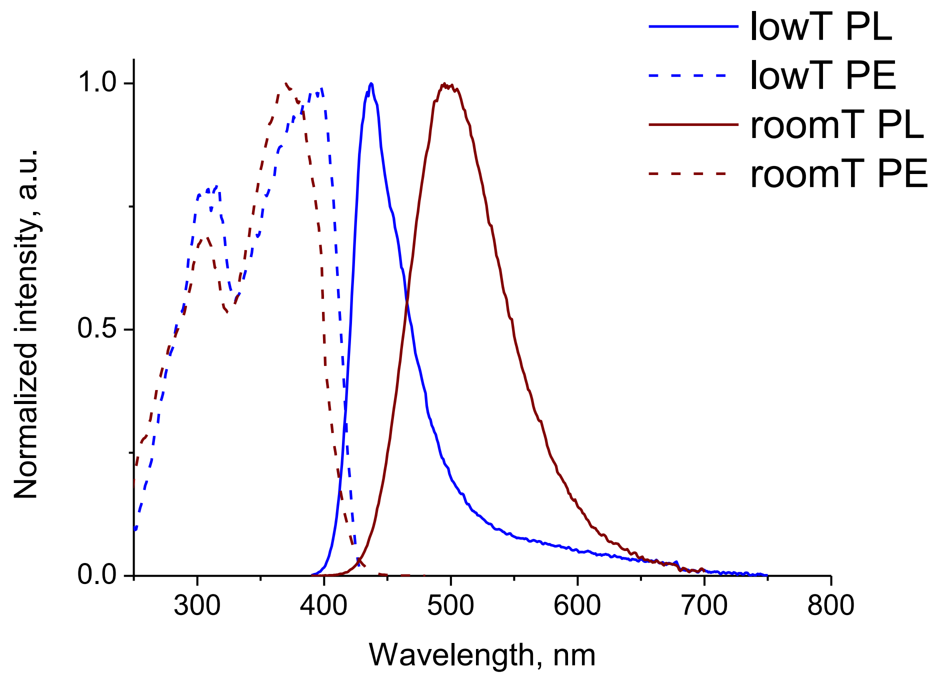

lowT PL
lowT PE
roomT PL
roomT PE
Normalized intensity, a.u.
1.0
0.5
0.0
300
400
500
600
700
800
Wavelength, nm

DMF
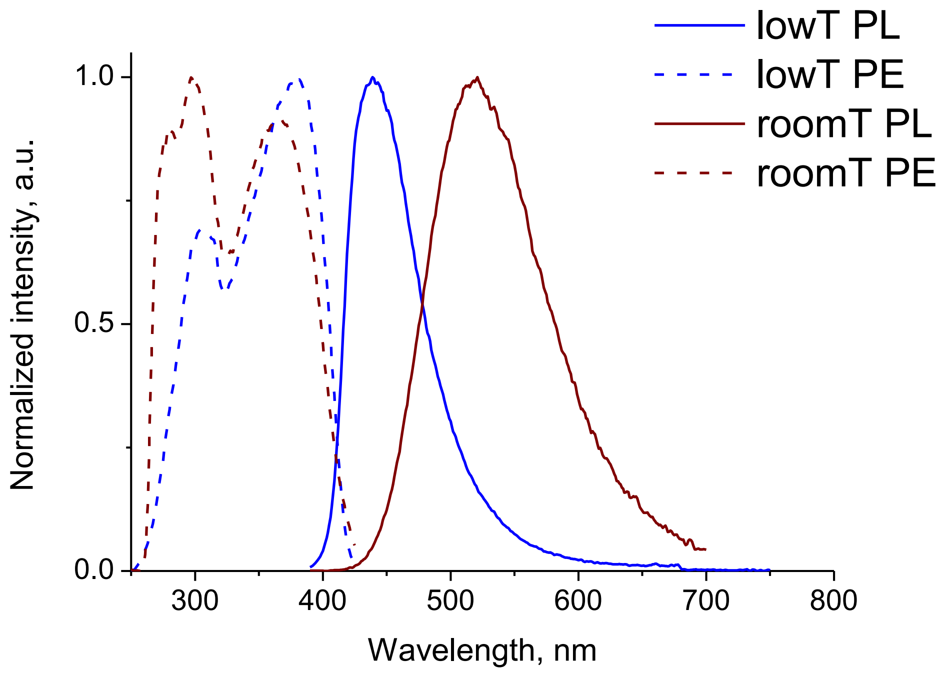 lowT PL<br>lowT PE<br>roomT PL<br>roomT PE<br>Normalized intensity, a.u.<br>0.0<br>0.5<br>1.0<br>300<br>400<br>500<br>600<br>700<br>800<br>Wavelength, nm
**DMSO**
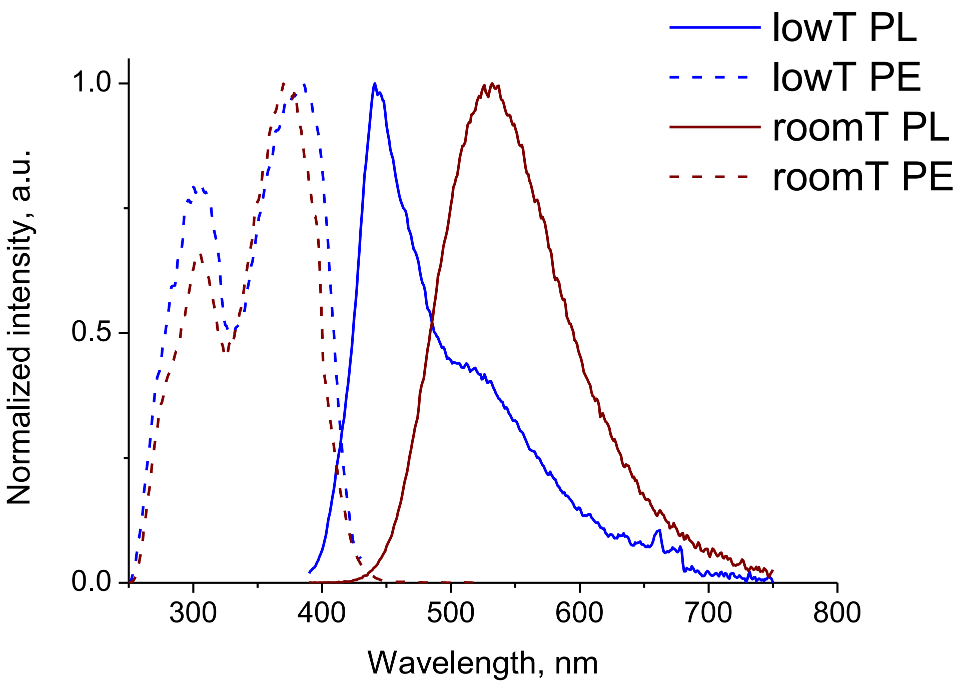 lowT PL<br>lowT PE<br>roomT PL<br>roomT PE<br>Normalized intensity, a.u.<br>0.0<br>0.5<br>1.0<br>300<br>400<br>500<br>600<br>700<br>800<br>Wavelength, nm

ACN

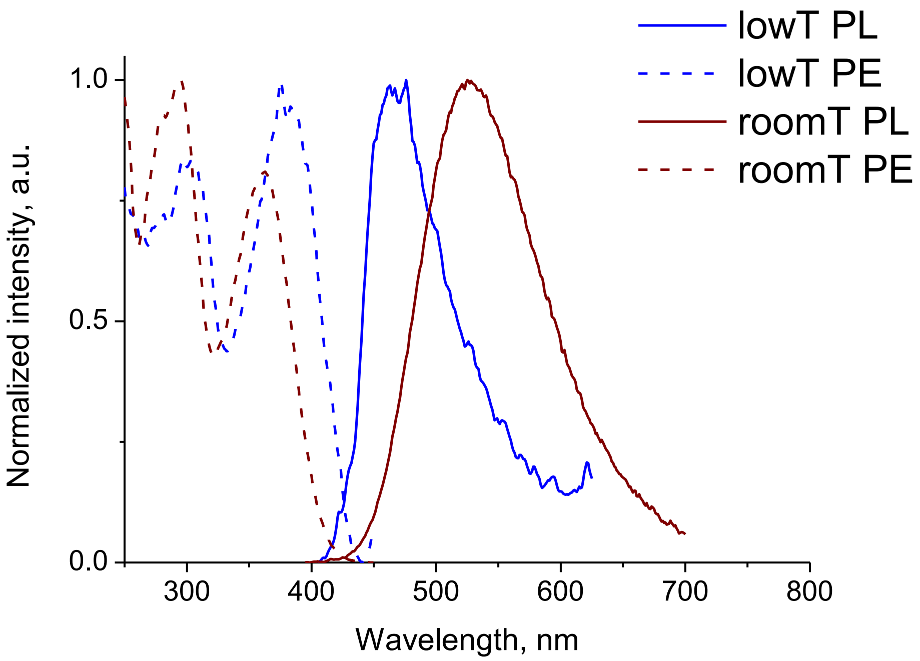

Figure S4. Comparison of roomT (purple) and 77 K (lowT, blue) photoexcitation (PE) and photoemission (PL) spectra of **tBuTPAterpy** in solvent of various polarity.

Time correlated single photon counting measurements

Photoluminescence lifetimes were measured with the time correlated single photon counting (TCSPC) method using a FLS-980 fluorescence spectrophotometer (Edinburgh Instruments) equipped with picosecond pulsed diodes (EPLED 375 nm) as excitation light sources and a photomultiplier (Hamamatsu, R928P, Japan) as detector. Prior to the analysis of a fluorescence decay, IRF measurements were performed at the selected excitation wavelength using a LUDOX® solution as standard. The FLS-980 software was used to fit the decay curves (Figure S5).

roomT

Hex

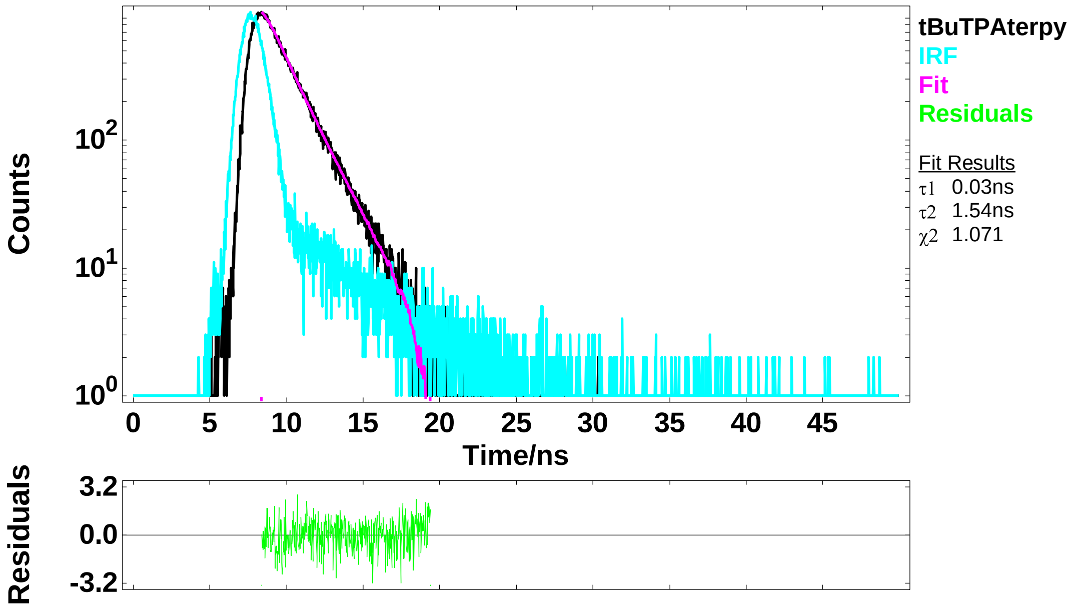

CHx

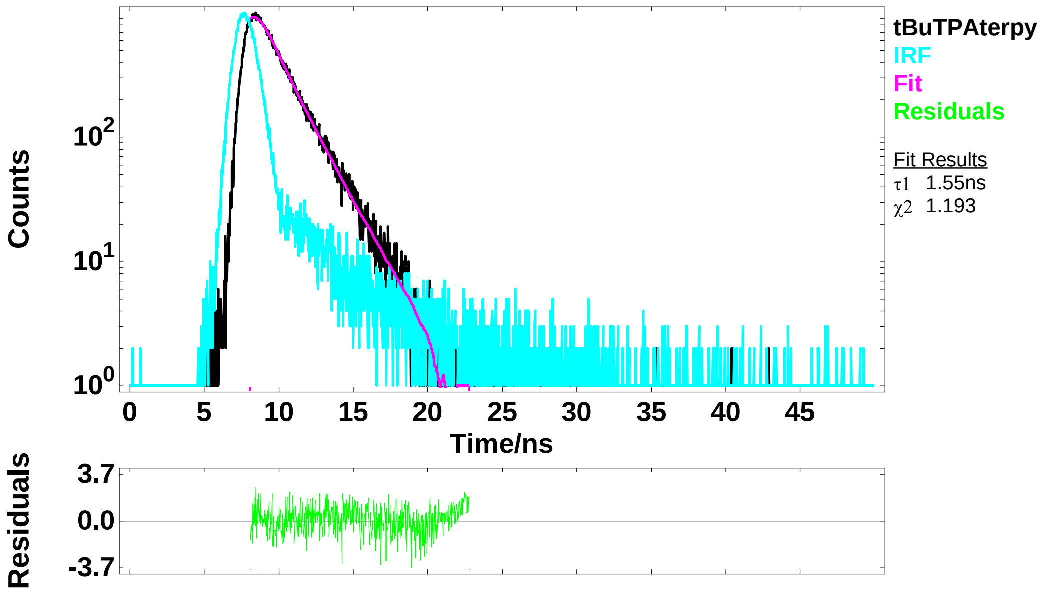

Tol

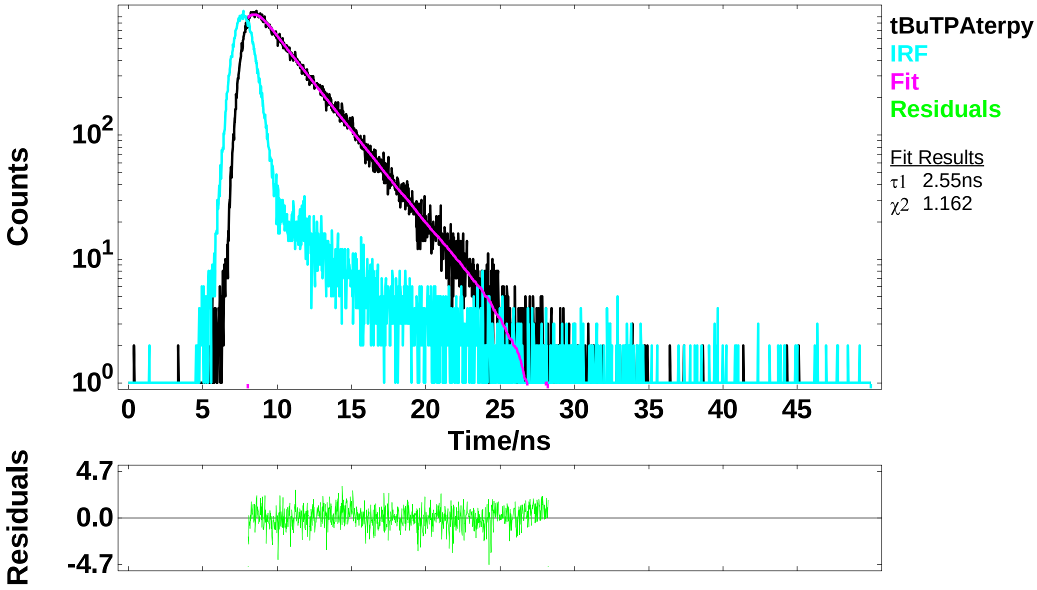
tBuTPAterpy
IRF
Fit
Residuals
Fit Results
τ1 2.55ns
χ2 1.162
Counts
Time/ns
Residuals

$CHCl_3$ [1]

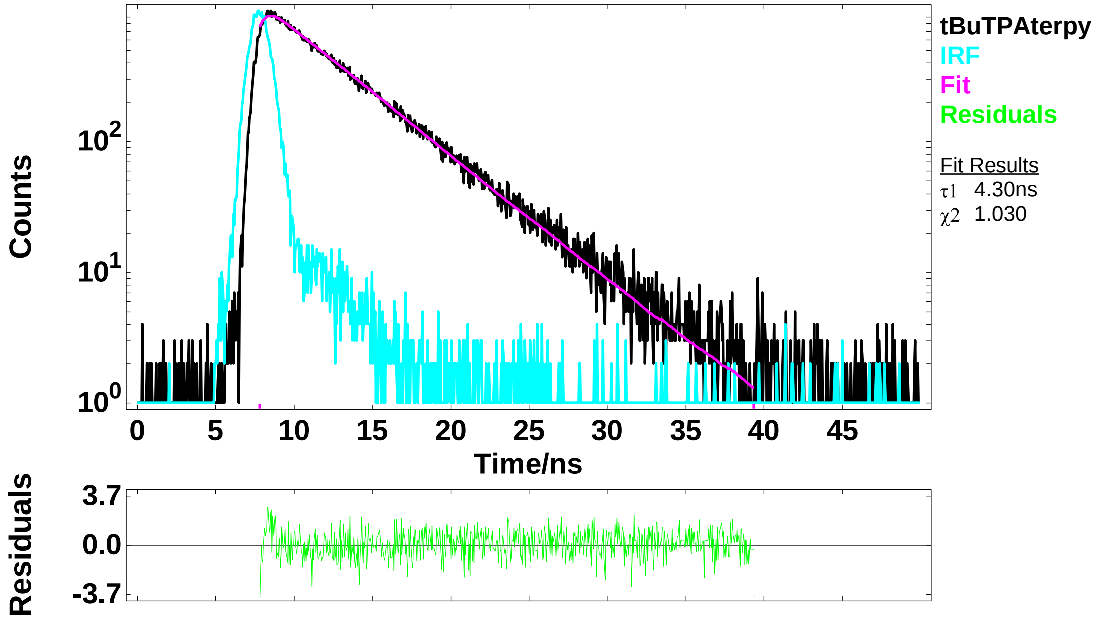
tBuTPAterpy
IRF
Fit
Residuals
Fit Results
τ1 4.30ns
χ2 1.030
Counts
Time/ns
Residuals

EtAc

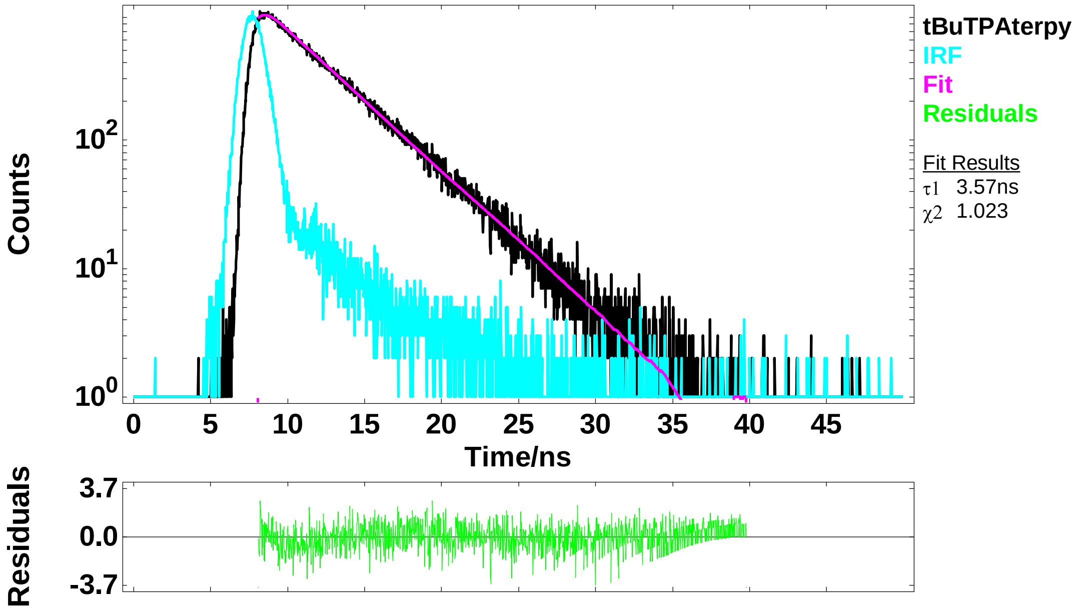
tBuTPAterpy
IRF
Fit
Residuals
Fit Results
τ1 3.57ns
χ2 1.023
Counts
10^2
10^1
10^0
0 5 10 15 20 25 30 35 40 45
Time/ns
Residuals
3.7
0.0
-3.7

THF

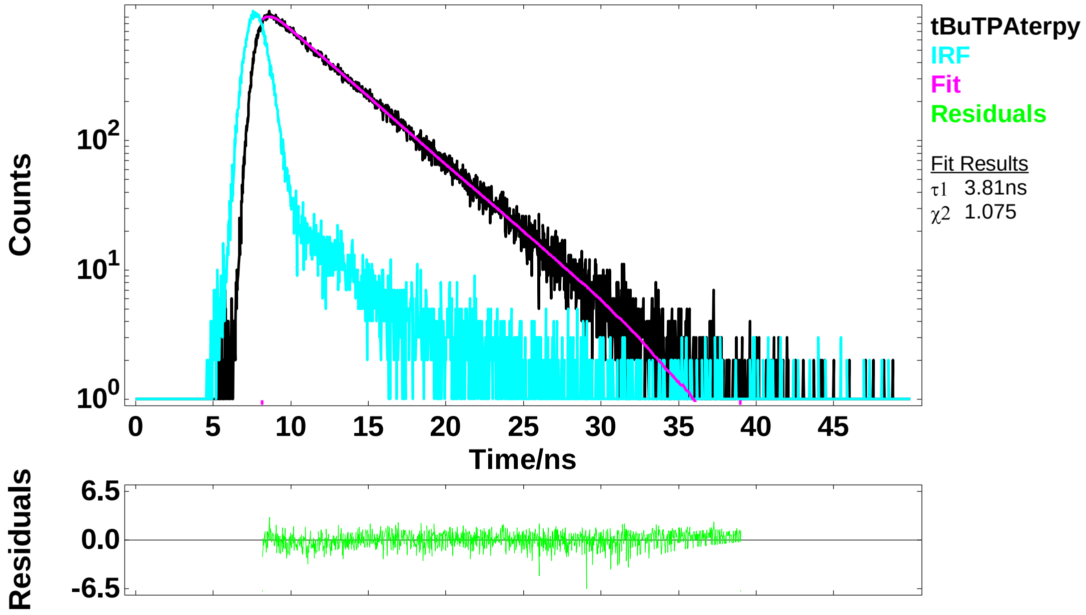
tBuTPAterpy
IRF
Fit
Residuals
Fit Results
τ1 3.81ns
χ2 1.075
Counts
10^2
10^1
10^0
0 5 10 15 20 25 30 35 40 45
Time/ns
Residuals
6.5
0.0
-6.5

DCM

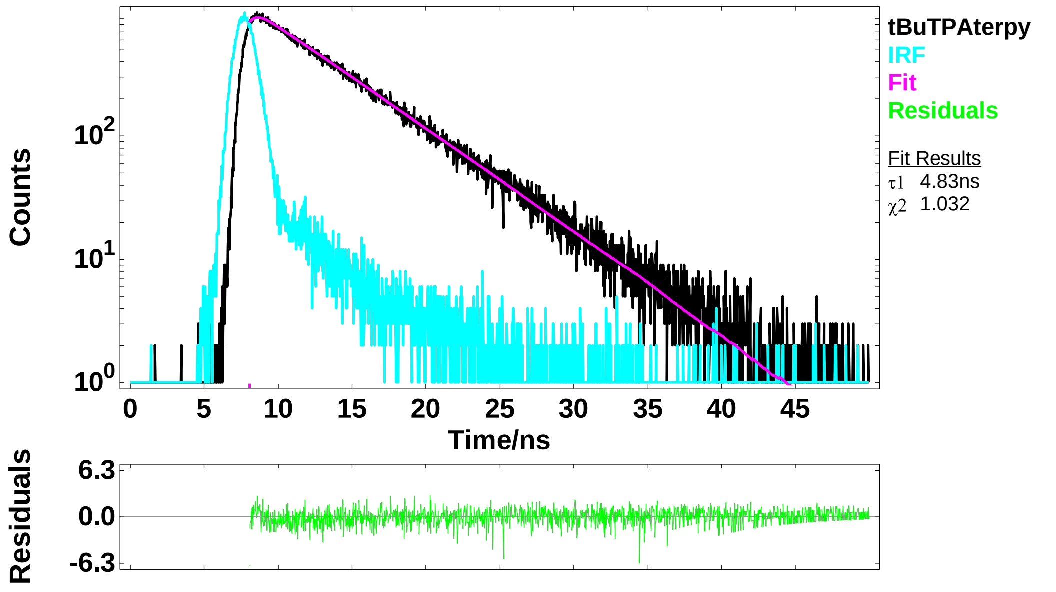

tBuTPAterpy
IRF
Fit
Residuals
Fit Results
τ1 4.83ns
χ2 1.032
Counts
10^2
10^1
10^0
0 5 10 15 20 25 30 35 40 45
Time/ns
Residuals
6.3
0.0
-6.3

DFM

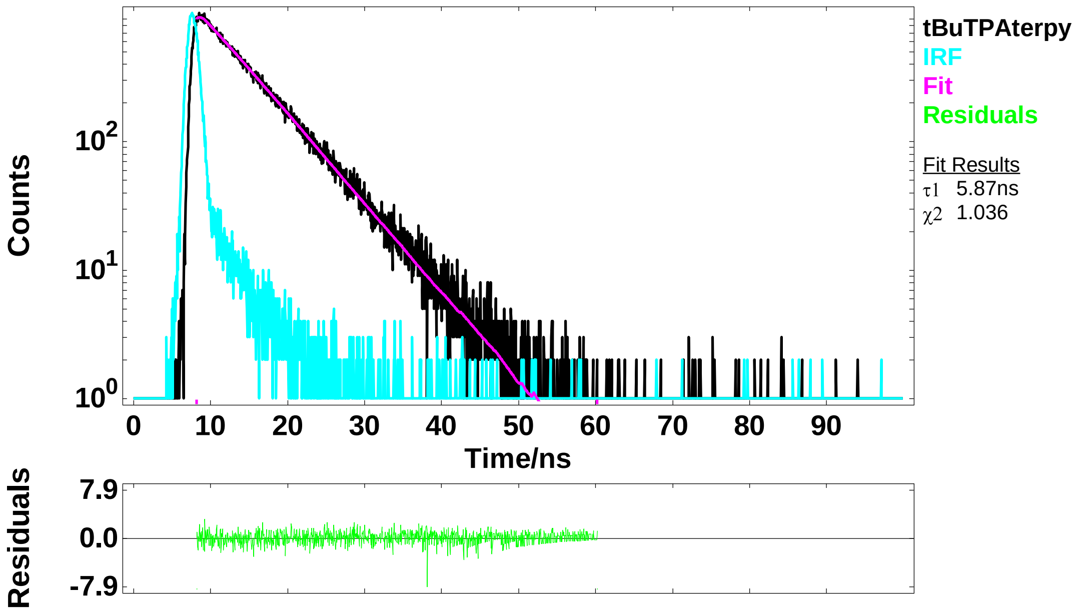

tBuTPAterpy
IRF
Fit
Residuals
Fit Results
τ1 5.87ns
χ2 1.036
Counts
10^2
10^1
10^0
0 10 20 30 40 50 60 70 80 90
Time/ns
Residuals
7.9
0.0
-7.9

DMSO

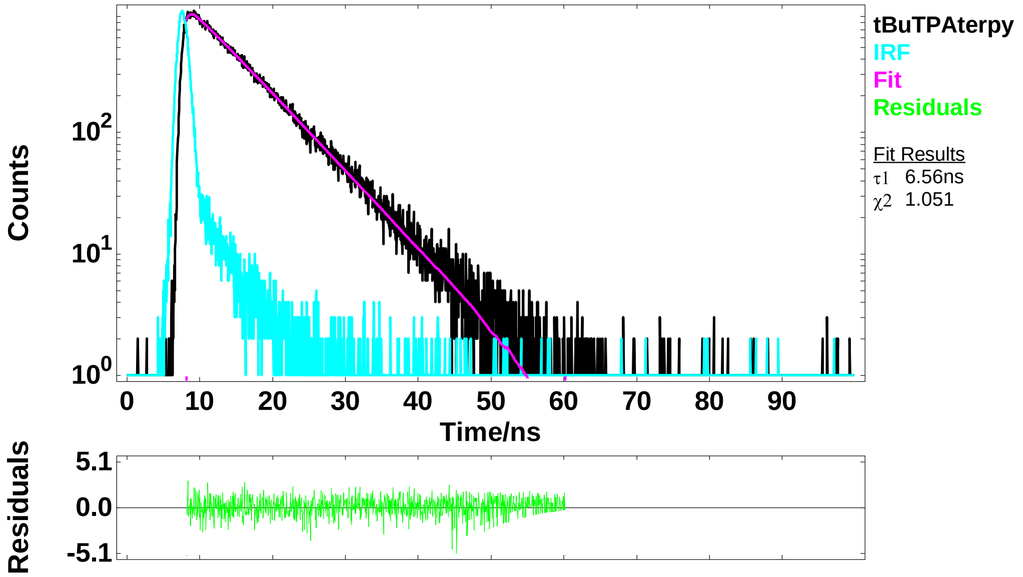

tBuTPAterpy
IRF
Fit
Residuals
Fit Results
τ1 6.56ns
χ2 1.051
Counts
Time/ns
Residuals

ACN [1]

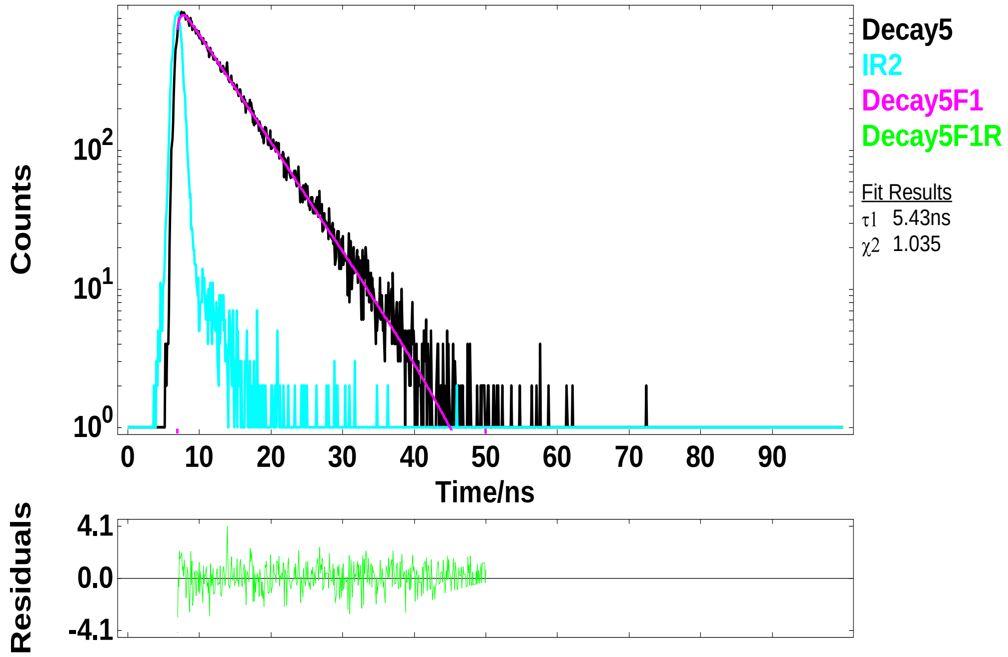

Decay5
IR2
Decay5F1
Decay5F1R
Fit Results
τ1 5.43ns
χ2 1.035
Counts
Time/ns
Residuals

BuCN

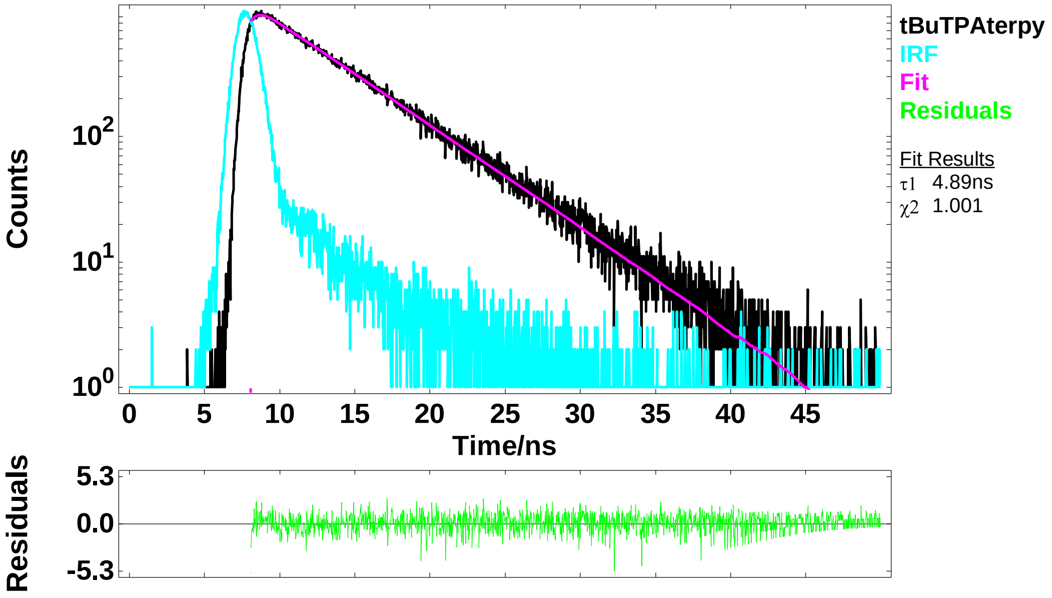
Counts
10^2
10^1
10^0
0 5 10 15 20 25 30 35 40 45
Time/ns
tBuTPAterpy
IRF
Fit
Residuals
Fit Results
τ1 4.89ns
χ2 1.001
Residuals
5.3
0.0
-5.3

77 K

Hex

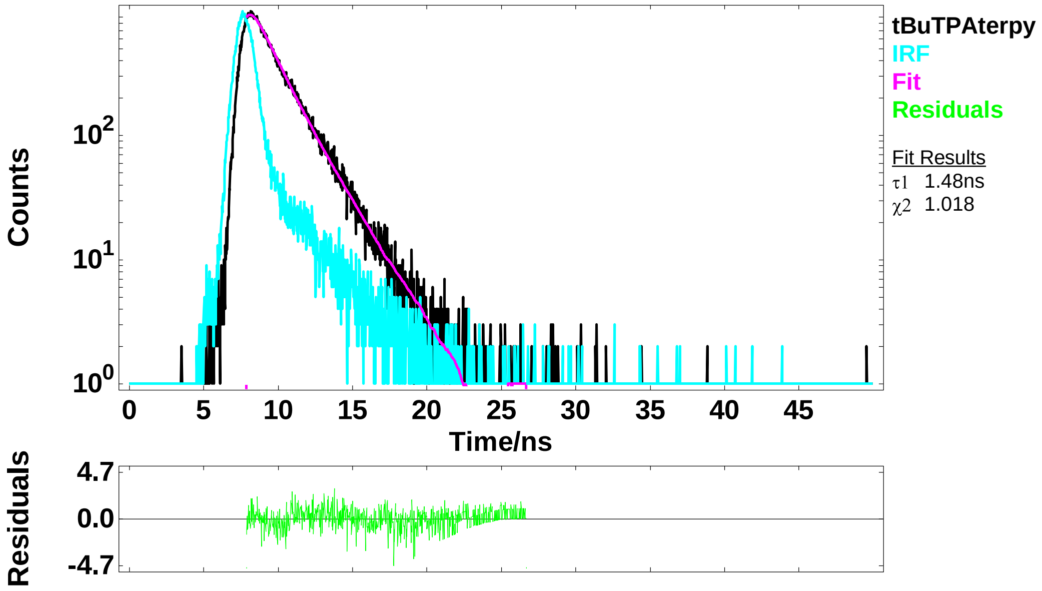
Counts
10^2
10^1
10^0
0 5 10 15 20 25 30 35 40 45
Time/ns
tBuTPAterpy
IRF
Fit
Residuals
Fit Results
τ1 1.48ns
χ2 1.018
Residuals
4.7
0.0
-4.7

CHx

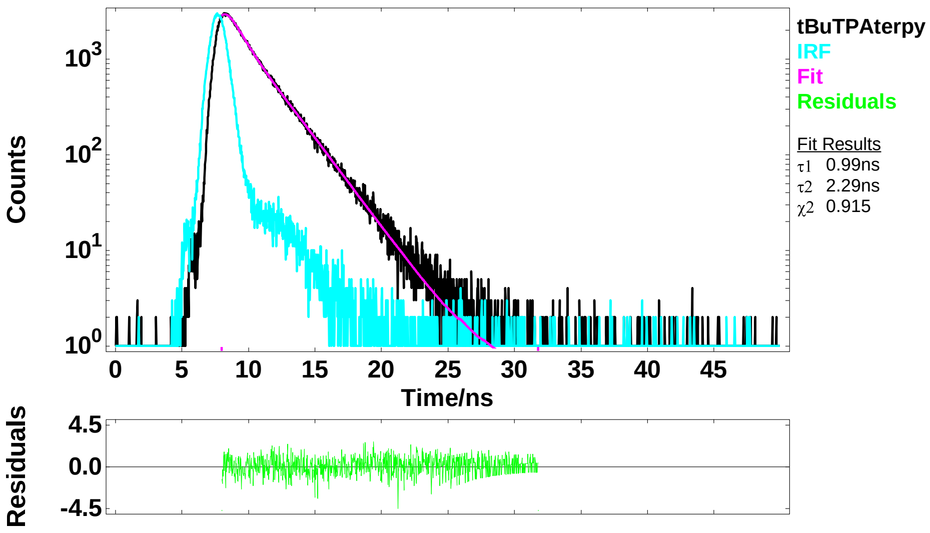

tBuTPAterpy
IRF
Fit
Residuals
Fit Results
τ1 0.99ns
τ2 2.29ns
χ2 0.915
Counts
Time/ns
Residuals

Tol

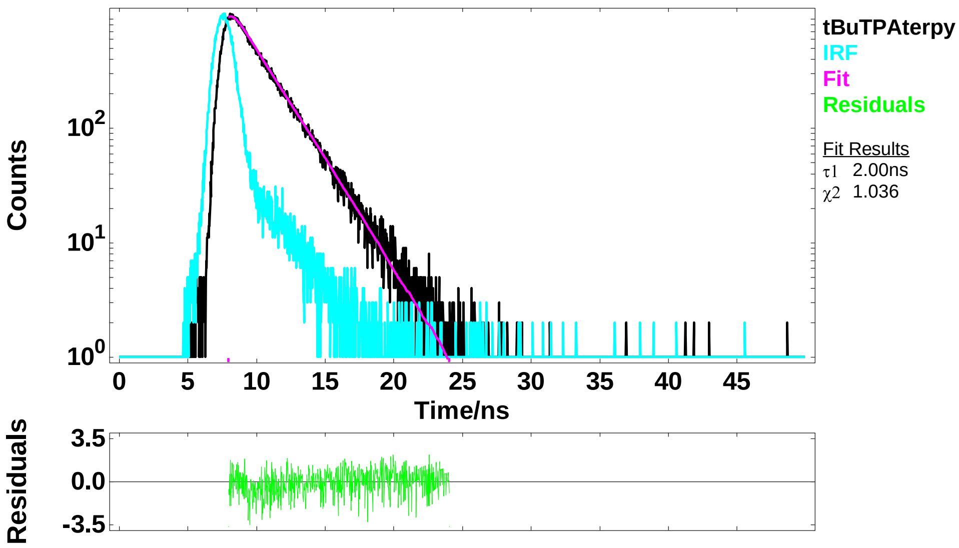

tBuTPAterpy
IRF
Fit
Residuals
Fit Results
τ1 2.00ns
χ2 1.036
Counts
Time/ns
Residuals

$CHCl_3$

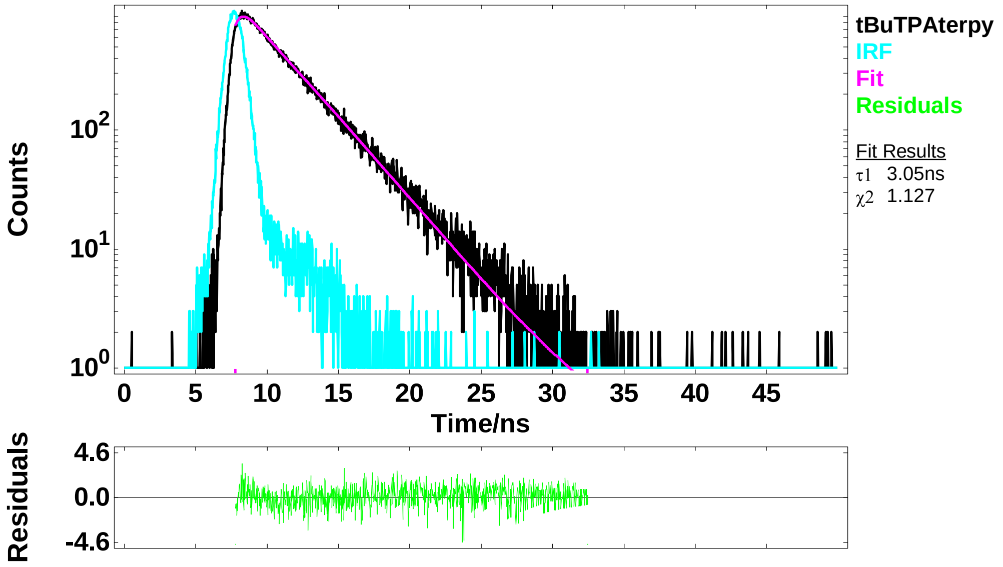
tBuTPAterpy
IRF
Fit
Residuals
Fit Results
τ1 3.05ns
χ2 1.127
Counts
Time/ns
Residuals
4.6
0.0
-4.6

EtAc

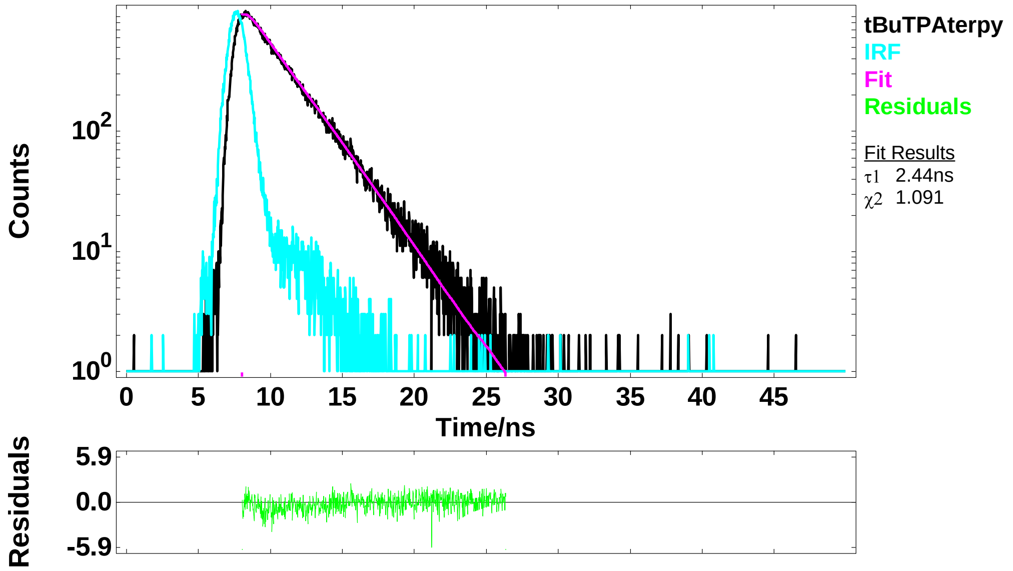
tBuTPAterpy
IRF
Fit
Residuals
Fit Results
τ1 2.44ns
χ2 1.091
Counts
Time/ns
Residuals
5.9
0.0
-5.9

THF

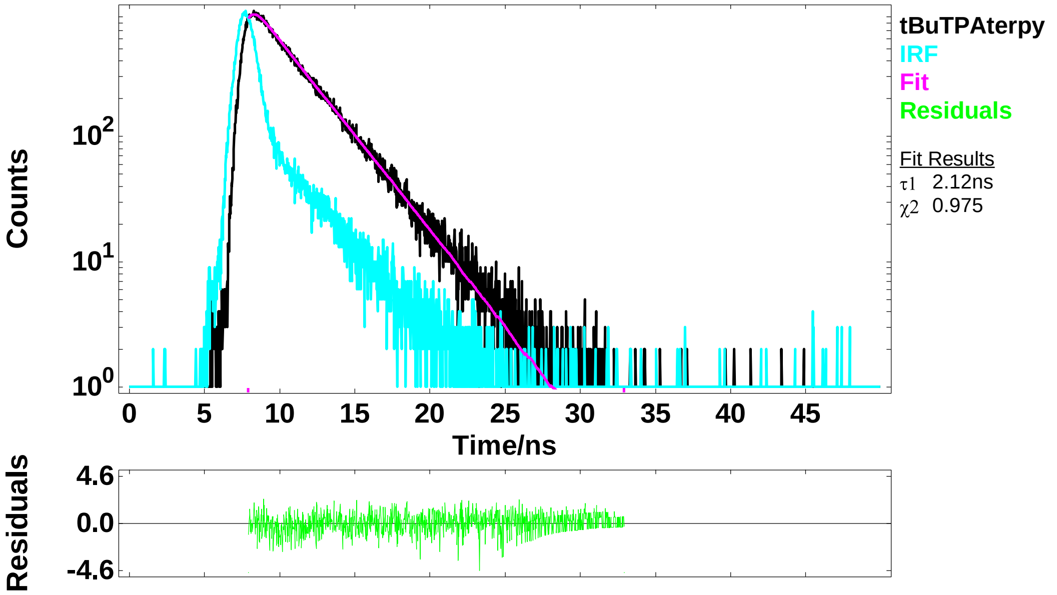

tBuTPAterpy
IRF
Fit
Residuals
Fit Results
τ1 2.12ns
χ2 0.975
Counts
Time/ns
Residuals

DCM

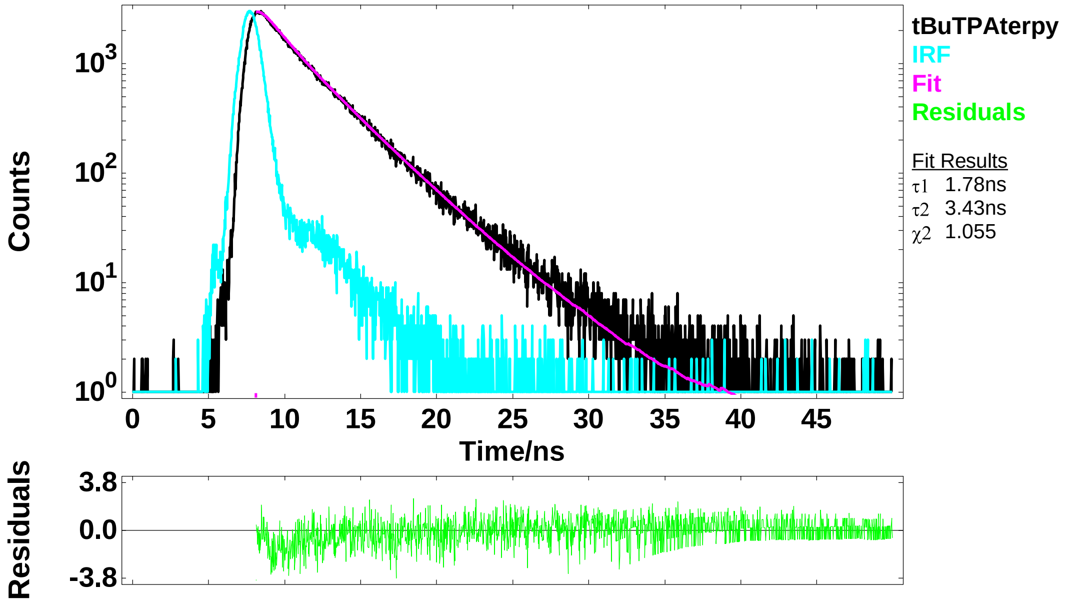

tBuTPAterpy
IRF
Fit
Residuals
Fit Results
τ1 1.78ns
τ2 3.43ns
χ2 1.055
Counts
Time/ns
Residuals

DMF

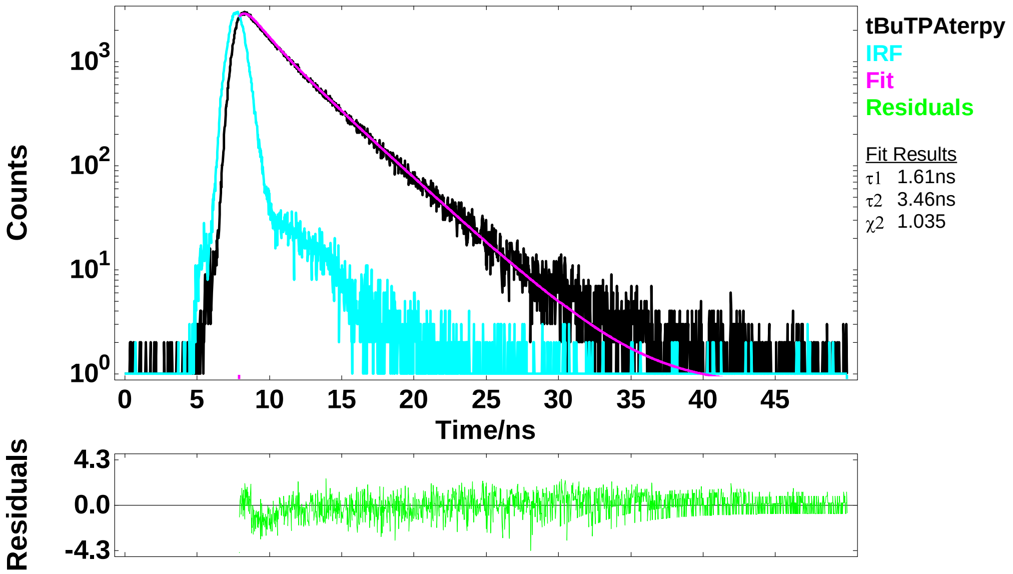
Counts
Residuals
Time/ns
tBuTPAterpy
IRF
Fit
Residuals
Fit Results
τ1 1.61ns
τ2 3.46ns
χ2 1.035

DMSO

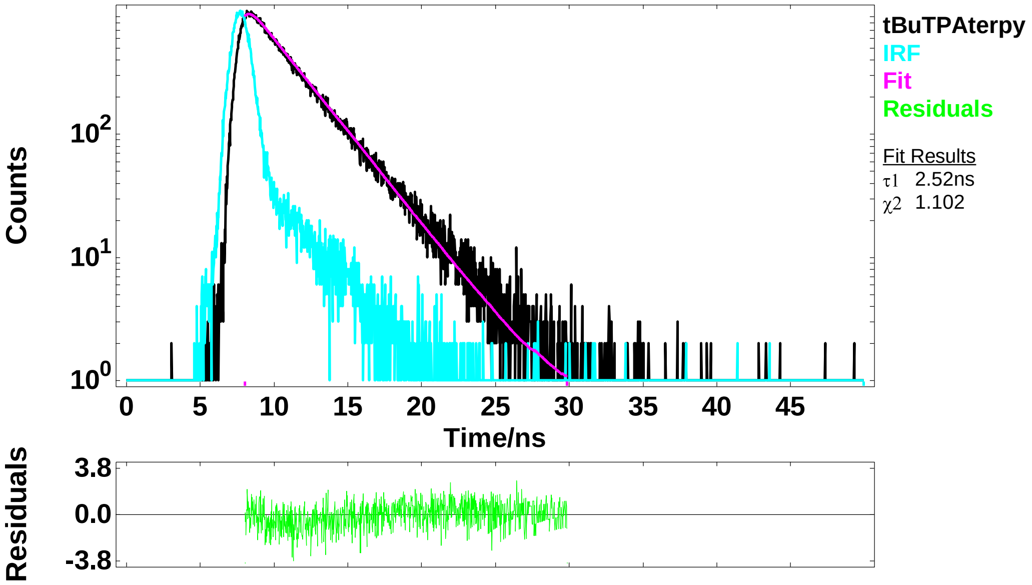
Counts
Residuals
Time/ns
tBuTPAterpy
IRF
Fit
Residuals
Fit Results
τ1 2.52ns
χ2 1.102

CH_3CN

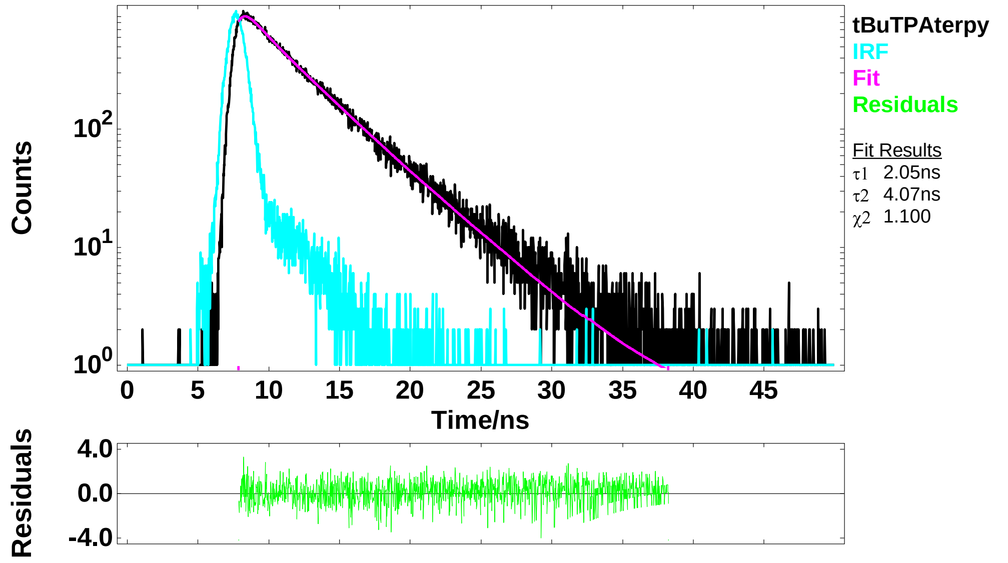

MeOH:EtOH

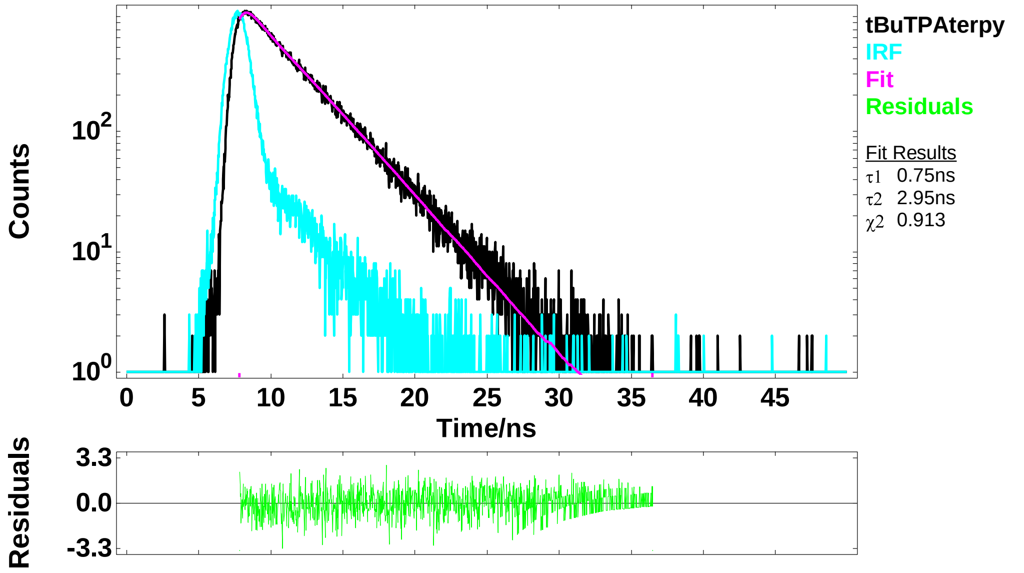

BuCN
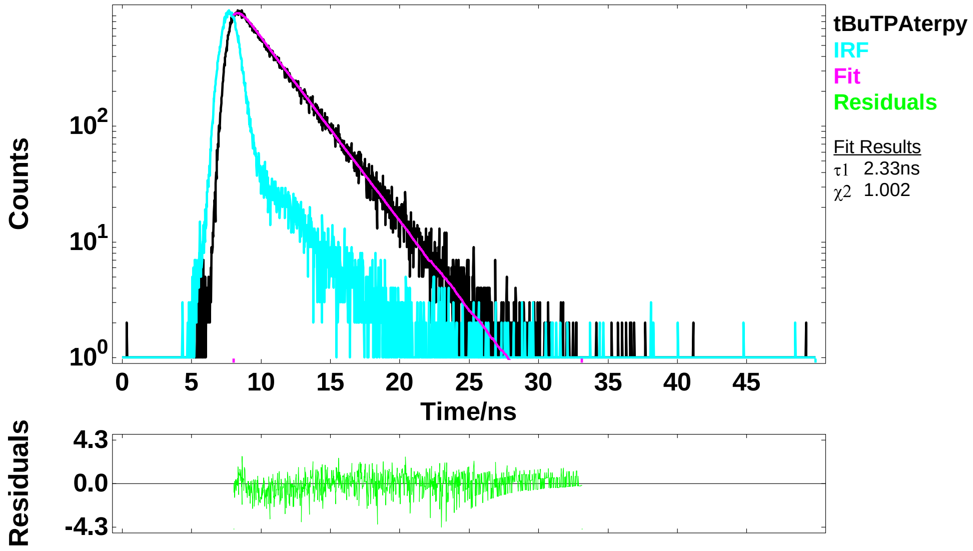

Figure S5. PL decay curves of **tBuTPAterpy** in various solvents.

Table S2. Spectral properties of **tBuTPAterpy** at room temperature.

Solvent	Δf	Viscosity (25°C, cP)	λ_{abs}, nm (ε, $10^3 \cdot M^{-1} cm^{-1}$)	λ_{PE}, nm	λ_{PL}, nm	Φ_{PL}	τ, ns	χ^2
Hexane	0.000	0.28[a]	364 (87.4), 291 (107.4)	365	407	0.48	1.53 ± 0.01	1.071
Cyclohexane	0.001	0.84[a]	366 (57.0), 293 (68.8)	365	408	0.54	1.55 ± 0.01	1.193
Toluene	0.013	0.52[a]	369 (74.6), 293 (96.7)	368	435	0.64	2.55 ± 0.01	1.162
Chloroform [1]	0.152	0.51[a]	371 (74.6), 294 (100.2)	371	487	0.84	4.30 ± 0.02	1.030
Ethyl acetate	0.201	0.40[a]	363 (74.1), 291 (92.2)	364	468	0.70	3.57 ± 0.01	1.023
THF	0.210	0.44[a]	364 (75.2), 291 (101.8)	365	467	0.75	3.81 ± 0.01	1.075
Dichloromethane	0.218	0.41[b]	369 (42.8), 292 (59.1)	367	497	0.79	4.83 ± 0.01	1.032
DMF	0.274	0.79[a]	367 (33.3), 291 (46.0)	365	518	0.77	5.87± 0.02	1.036
DMSO	0.263	1.99[c]	372 (46.8), 292 (67.4)	372	528	0.83	6.56± 0.02	1.051
Acetonitrile [1]	0.305	0.34[c]	364 (31.6), 289 (41.5)	363	527	0.63	5.53± 0.03	1.165
Methanol	0.308	0.54[b,c]	369 (47.3), 290 (66.6)	371	557	0.02	n.d.	n.d.

a. [2]

b. [3]

c. [4]

Table S3. Spectral properties of **tBuTPAterpy** at 77 K.

Solvent	λ_{PE}, nm	λ_{PL}, nm	τ, ns	χ^2
Hexane	403	438	1.48 ± 0.01	1.018
Cyclohexane	385	418	0.99 ± 0.06 (24.74%),<br>2.29 ± 0.02 (74.26%)	0.915
Toluene	396	422	2.00 ± 0.01	1.036
Chloroform	394	442	3.05 ± 0.01	1.127
Ethyl acetate	387	424	2.44 ± 0.01	1.091
THF	397	428	2.12 ± 0.01	0.975
Dichloromethane	395	439	1.78 ± 0.06 (41.12%),<br>3.43 ± 0.05 (58.88%)	1.055
DMF	380	439	1.61 ± 0.06 (30%),<br>3.46 ± 0.03 (70%)	1.035
DMSO	386	441, 516sh	2.52 ± 0.01	1.102
Acetonitrile	383	463	2.05 ± 0.20 (27.77%),<br>4.07 ± 0.12 (72.23%)	1.100
Methanol:Ethanol	394	438	2.95 ± 0.02<br>0.75 ± 0.23	0.913
Butyronitrile (77 K)	387	429	2.33 ± 0.01	1.002
Butyronitrile (roomT)	367	508	4.89 ± 0.01	1.001

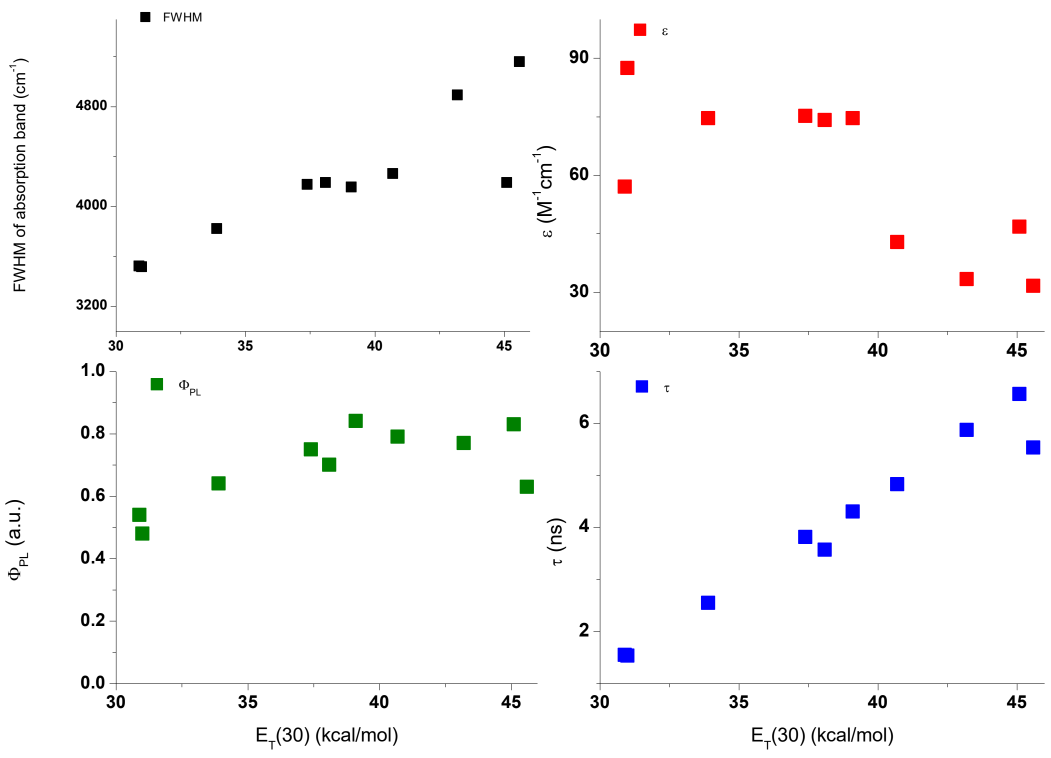

Figure S6. Solvent polarity $E_T(30)$ (kcal·mol^{-1}) dependence of: absorption band FWHM (cm^{-1}) (black), extinction coefficient of the absorption maximum (M^{-1} cm^{-1}) (red), photoluminescence quantum yield (a.u.) (green) and lifetime (ns) (blue).

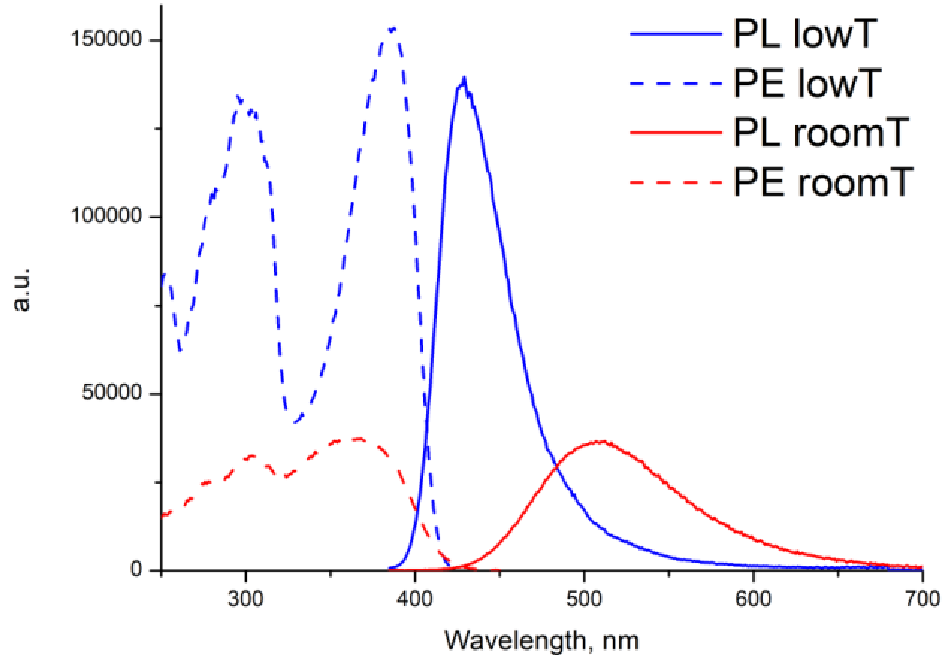

a

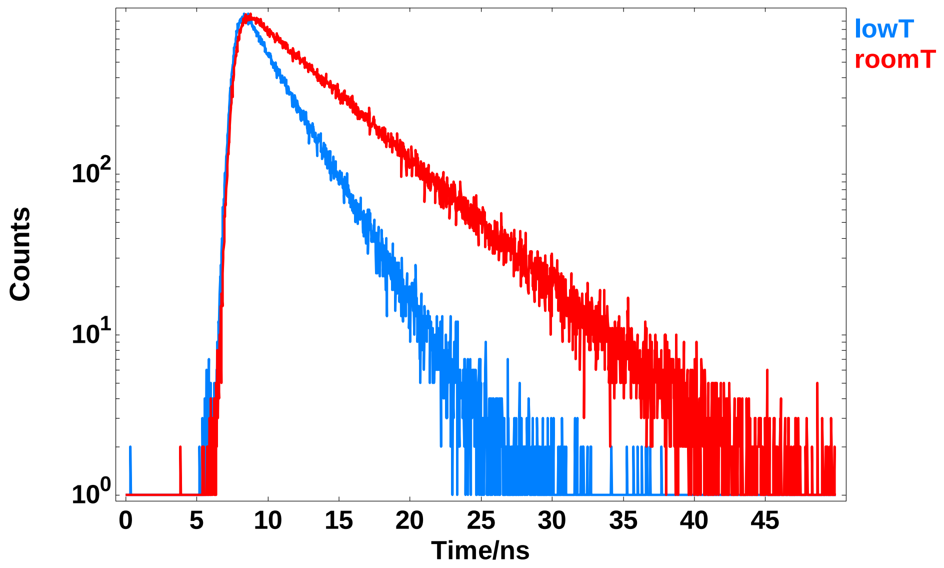

b

Figure S7. (a) tBuTPAterpy emission intensity and **(b)** PL decay time in BuCN at room temperature (roomT, red) and 77 K (lowT, blue).

Time resolved emission maps

The TRES maps were obtained by measuring a series of TCSPC traces in the ranges 390-700 nm (at room temperature) and 390-630 nm (at 77 K) with a wavelength step of 5 nm and an excitation wavelength of 375 nm. The time windows were defined up to 100 ns. The pulse repetition period was set as 1μs. Prior to the measurements, the optimisation of the signal rate on the PMT and the time conditions was performed for the emission maximum wavelength obtained from steady-state experiments. The signal rate on PMT was set between 2000 - 6000 cps. Each decay trace was collected for 300 s (time stop conditions). All TRES maps are reported in Figure S8.

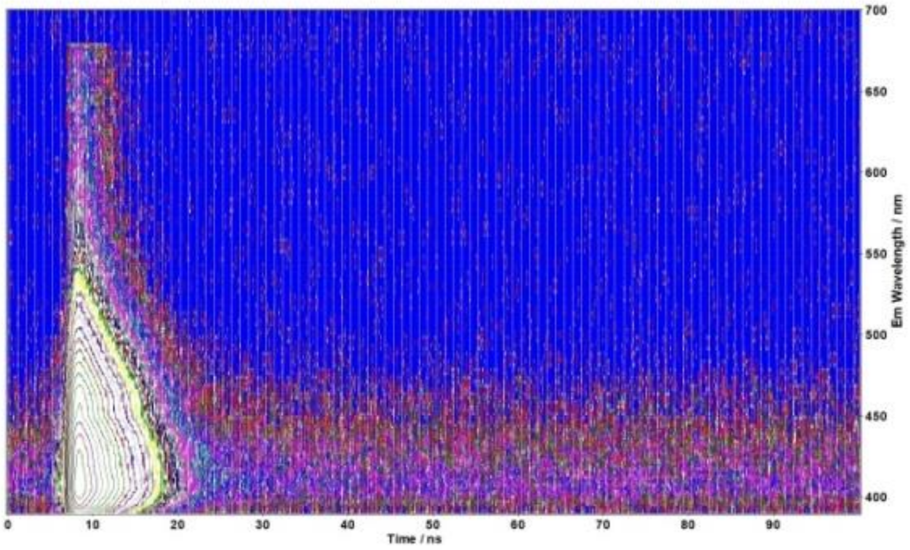

Hexane

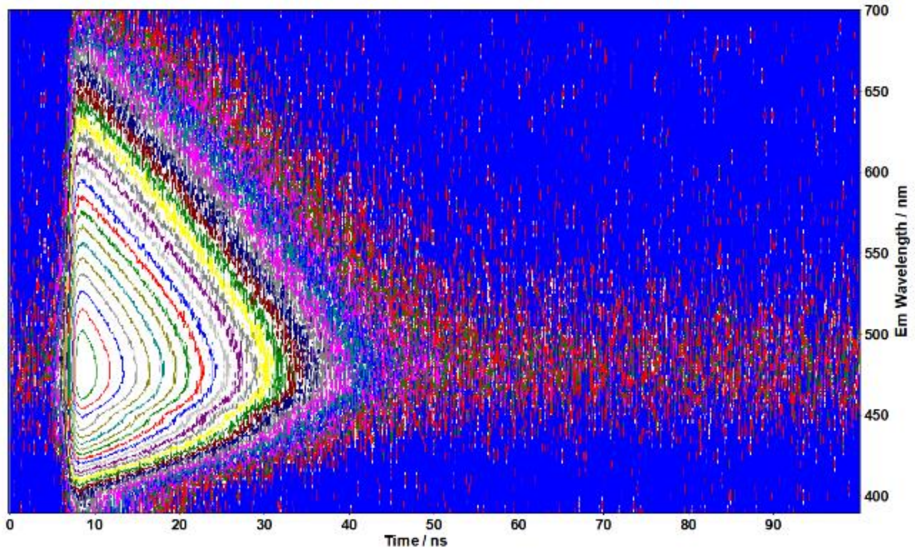

Chloroform

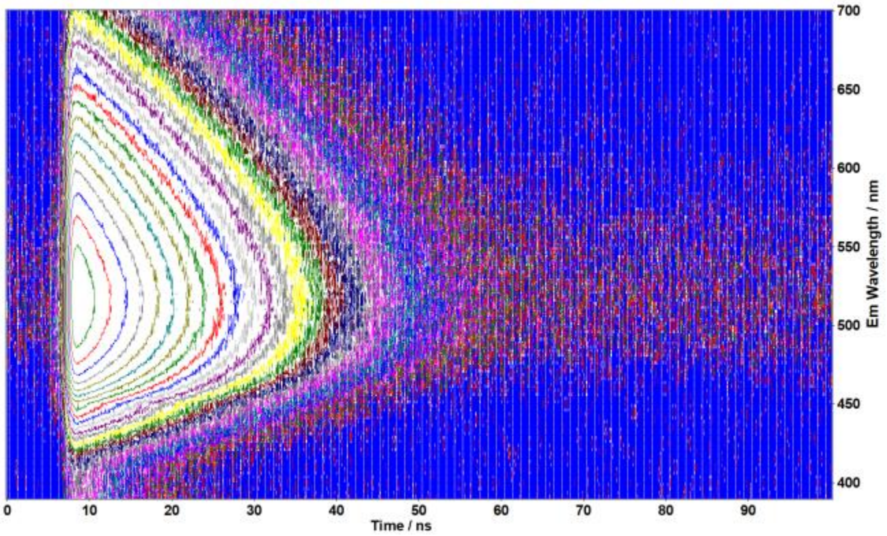

Acetonitrile

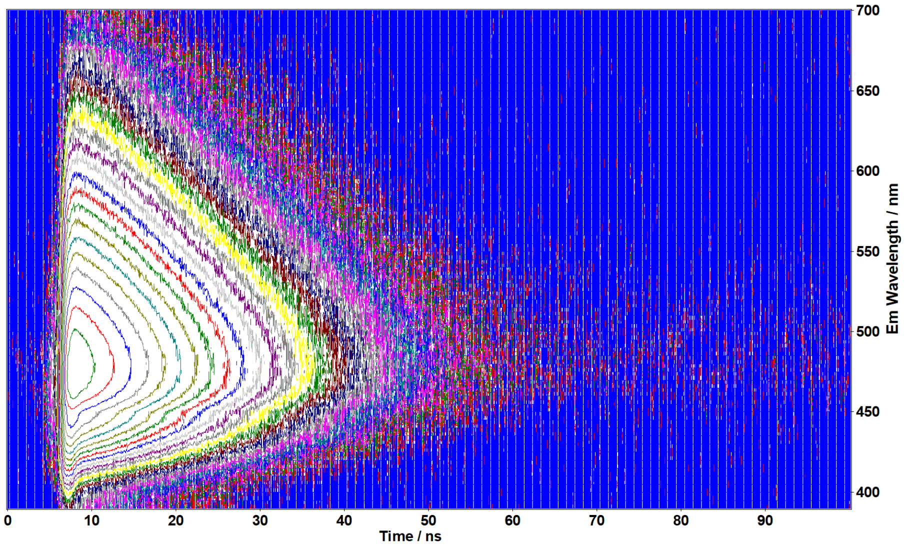

Glyceryl triacetate

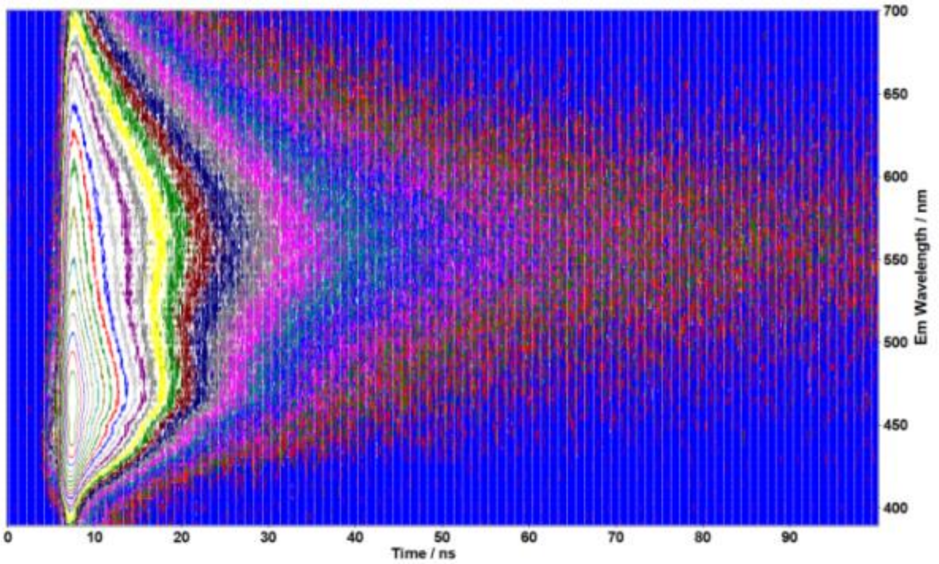

Glycerol

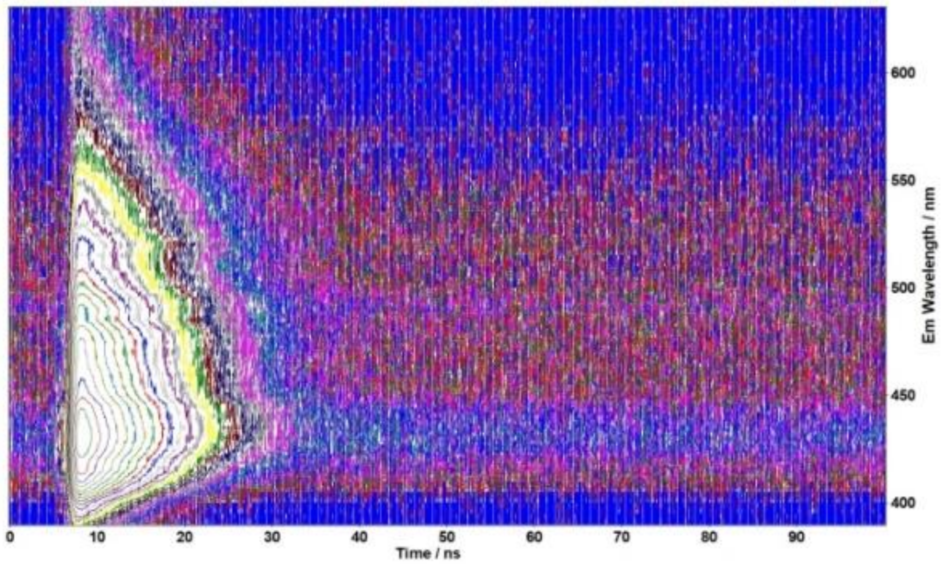

77 K (BuCN)

Figure S8. TRES maps of **tBuTPAterpy** in various solvents and temperatures.

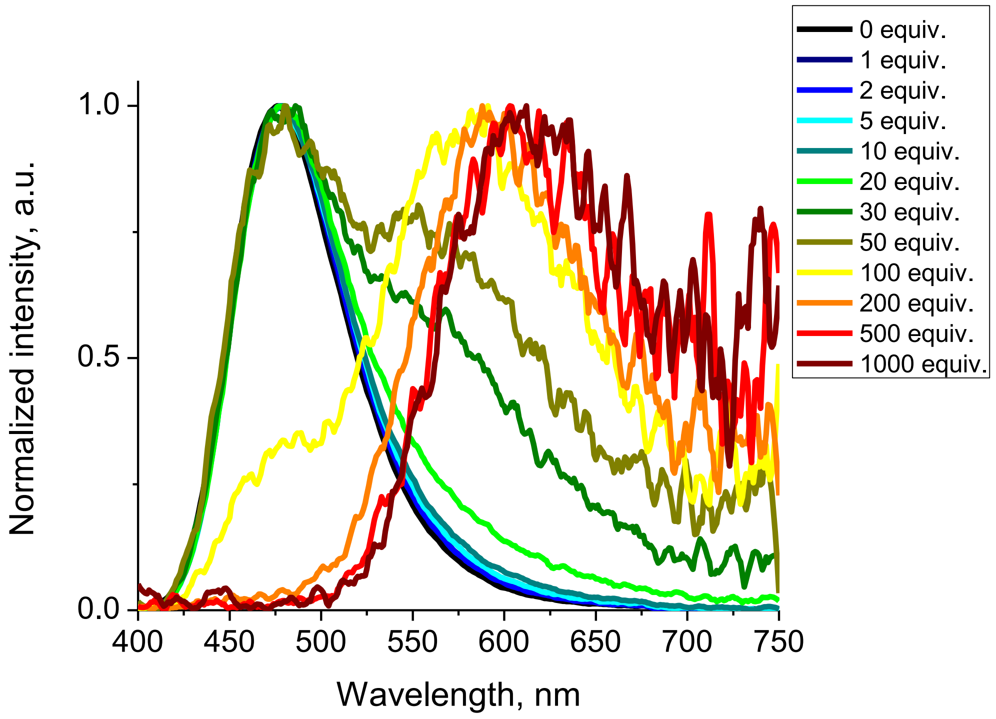

a

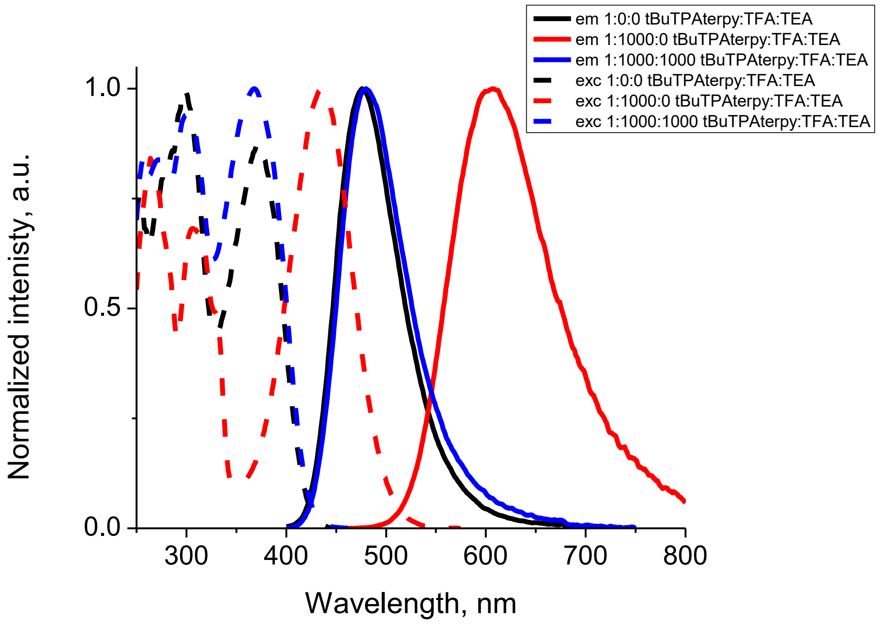

b

Figure S9. (a) Normalized PL spectra as a function of the titration of a **tBuTPAterpy** (1×10^{-5} M) chloroform solution with trifluoroacetic acid (TFA) (1-1000 equivalents)**. (b)** Normalized excitation (dash line) and emission (solid line) spectra of neutral (black), protonated (red) and deprotonated (blue) forms of **tBuTPAterpy**.

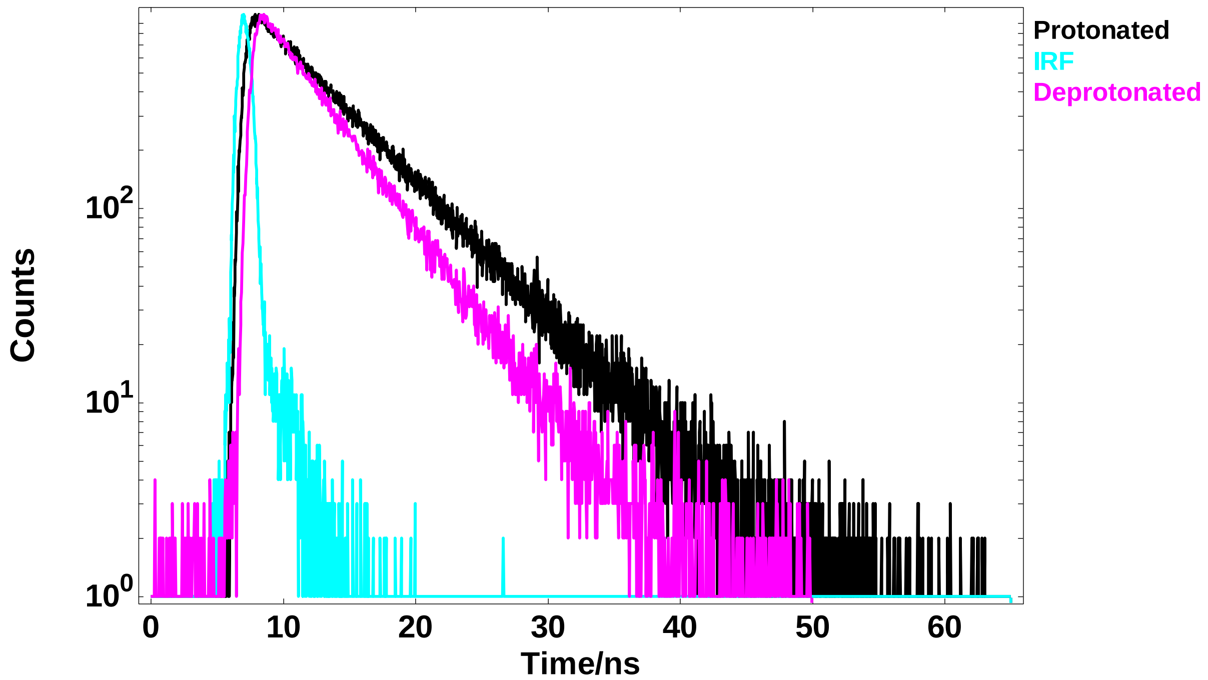

Figure S10. Comparison of the **tBuTPAterpy** decay curves for the protonated (black) and neutral (pink) form.

DFT calculations

Table S4. tBuTPAterpy five lowest singlet electronic excitation and the $S_1 \rightarrow S_0$, $T_1 \rightarrow S_0$ de-excitation energies.

	E [eV]	λ [nm]	*f*	%	Character	
			Hexane			
S_1	3.21	386.6	0.6820	96	H → L	$\pi_{R1/Ph} \rightarrow \pi^*_{R2/Ph}$
	3.08 [a)]	402.3 [a)]				
S_2	3.33	372.8	0.0001	98	H → L+1	$\pi_{R1/Ph} \rightarrow \pi^*_{R2}$
S_3	3.89	318.8	0.0265	84	H → L+3	$\pi_{R1/Ph} \rightarrow \pi^*_{R1/Ph}$
S_4	4.05	306.1	0.2844	73	H → L+2	$\pi_{R1/Ph} \rightarrow \pi^*_{R2/Ph}$
				17	H → L+5	$\pi_{R1/Ph} \rightarrow \pi^*_{R2}$
S_5	4.08	303.9	0.3364	97	H → L+6	$\pi_{R1/Ph} \rightarrow \pi^*_{R1}$
S_6	4.19	296.2	0.2926	89	H-1 → L	$\pi_{R2} \rightarrow \pi^*_{R2/Ph}$
**S_1**	2.62	473.6	0.2387	97	H → L	$\pi_{R1/Ph} \rightarrow \pi^*_{R2/Ph}$
	2.41 [a)]	515.5 [a)]				
	2.70 [b)]	458.8 [b)]				
**T_1**	2.22	558.2				
			Chloroform			
S_1	3.18	390.4	0.7025	96	H → L	$\pi_{R1/Ph} \rightarrow \pi^*_{R2/Ph}$
	3.01 [a)]	411.9 [a)]				
S_2	3.30	375.8	0.0001	98	H → L+1	$\pi_{R1/Ph} \rightarrow \pi^*_{R2}$
S_3	3.89	318.4	0.0287	81	H → L+3	$\pi_{R1/Ph} \rightarrow \pi^*_{R1/Ph}$
S_4	4.05	306.5	0.2888	74	H → L+2	$\pi_{R1/Ph} \rightarrow \pi^*_{R2/Ph}$
S_5	4.09	302.8	0.3306	97	H → L+6	$\pi_{R1/Ph} \rightarrow \pi^*_{R1}$
S_6	4.18	296.7	0.3073	90	H-1 → L	$\pi_{R2} \rightarrow \pi^*_{R2/Ph}$
**S_1**	2.65	467.5	0.4682	97	H → L	$\pi_{R1/Ph} \rightarrow \pi^*_{R2/Ph}$
	2.20 [a)]	564.5 [a)]				
	2.56 [b)]	483.9 [b)]				
**T_1**	2.20	564.3				
			Acetonitrile			
S_1	3.17	391.4	0.6997	96	H → L	$\pi_{R1/Ph} \rightarrow \pi^*_{R2/Ph}$
	3.01 [a)]	412.5 [a)]				
S_2	3.29	377.3	0.0001	98	H → L+1	$\pi_{R1/Ph} \rightarrow \pi^*_{R2}$
S_3	3.90	317.8	0.0281	84	H → L+3	$\pi_{R1/Ph} \rightarrow \pi^*_{R1/Ph}$
S_4	4.05	305.9	0.2921	75	H → L+2	$\pi_{R1/Ph} \rightarrow \pi^*_{R2/Ph}$
S_5	4.12	301.1	0.3008	97	H → L+6	$\pi_{R1/Ph} \rightarrow \pi^*_{R1}$
S_6	4.18	296.5	0.2954	89	H-1 → L	$\pi_{R2} \rightarrow \pi^*_{R2/Ph}$
**S_1**	2.64	469.5	0.6430	97	H → L	$\pi_{R1/Ph} \rightarrow \pi^*_{R2/Ph}$

	2.02 [a]	615.1 [a]
	2.46 [b]	503.2 [b]
**T_1**	2.17	571.1

[a] results obtained performing state-specific non-equilibrium PCM simulations.
[b] results obtained performing state-specific non-equilibrium PCM simulations for the computation of the adiabatic de-excitation energy.

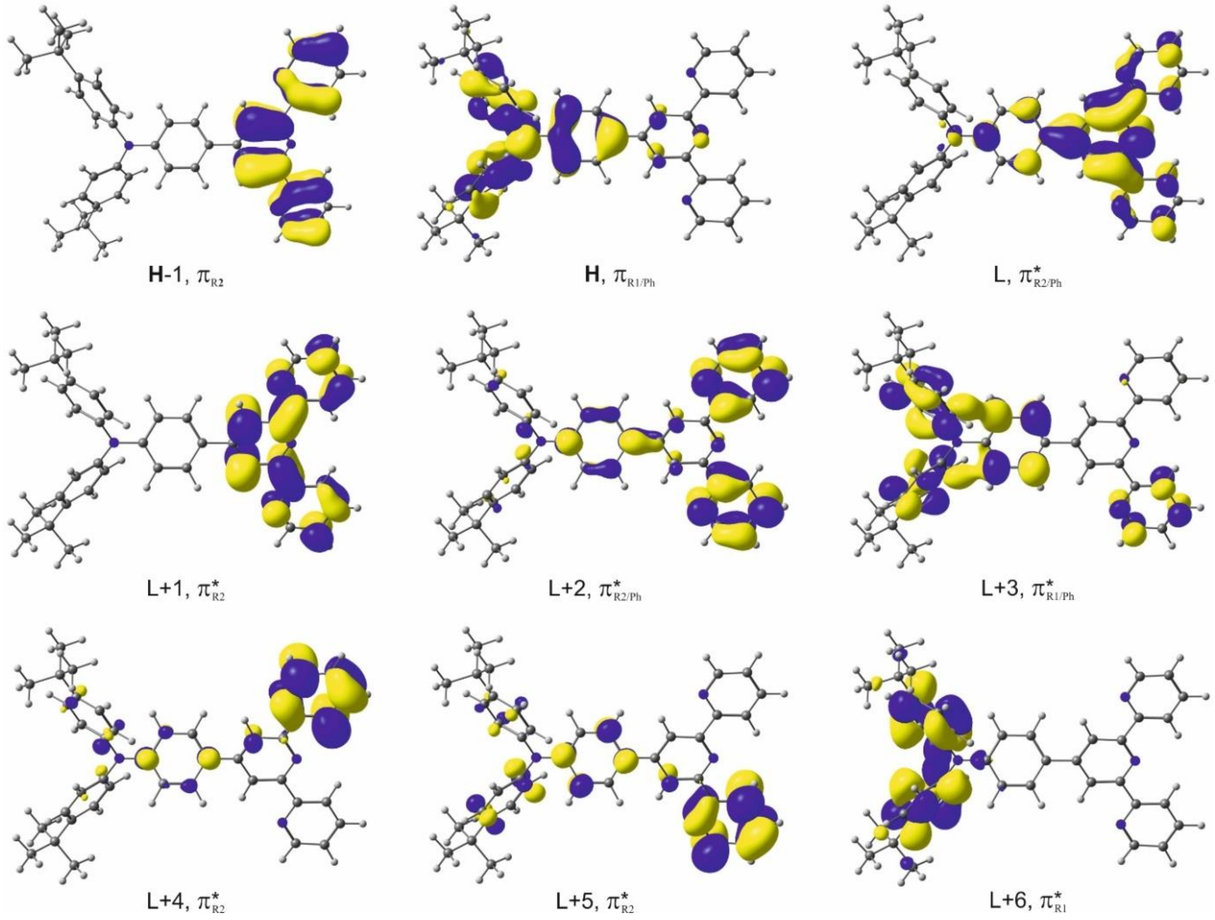

Figure S11. Kohn - Sham orbitals involved in six lowest electronic excitations for **tBuTPAterpy**. The character of the individual orbitals and the energy order are almost independent of the type of solvent used in the PCM model.

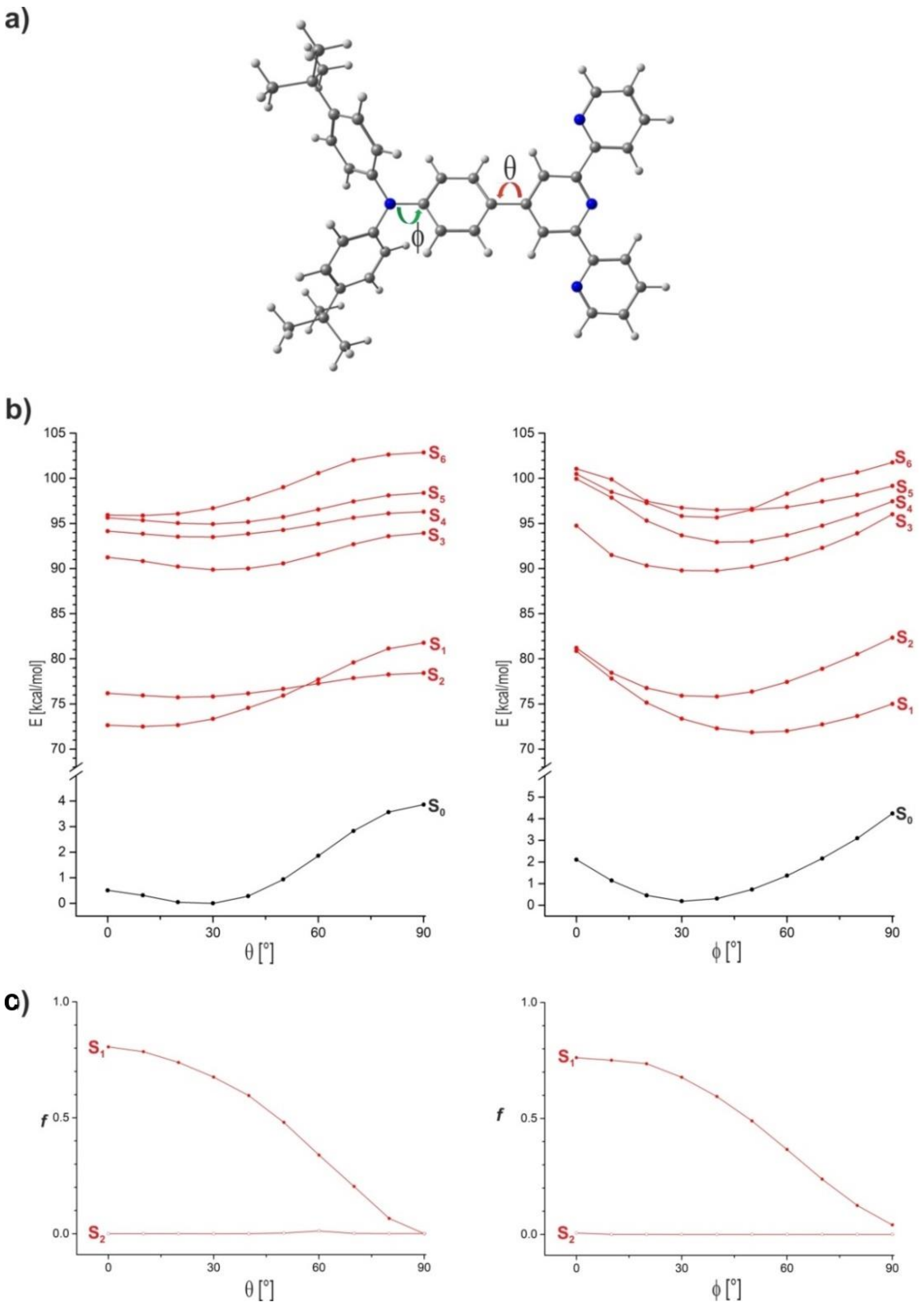

Figure S12. (a) Rotation angles between the central phenyl ring and the terpyridine (θ) and amino (ϕ) substituents, respectively. **(b)** Potential energy curves for S_0 ground state and the two lowest excited electronic states S_1 and S_2 as function of θ and ϕ dihedral angles. **(c)** Dependence of the oscillator strength on the rotation angle θ and ϕ for the singlet transitions S_1 and S_2. All simulations were preformed using the ACN solvent in the PCM model.

References

[1] Maroń AM, Szlapa-Kula A, Matussek M, Kruszynski R, Siwy M, Janeczek H, et al. Photoluminescence enhancement of Re(I) carbonyl complexes bearing D–A and D–π–A ligands. Dalton Trans 2020;49:4441–53. https://doi.org/10.1039/C9DT04871E.

[2] Jarusuwannapoom T, Hongrojjanawiwat W, Jitjaicham S, Wannatong L, Nithitanakul M, Pattamaprom C, et al. Effect of solvents on electro-spinnability of polystyrene solutions and morphological appearance of resulting electrospun polystyrene fibers. Eur Polym J 2005;41:409–21. https://doi.org/10.1016/j.eurpolymj.2004.10.010.

[3] Dong T, Knoshaug EP, Pienkos PT, Laurens LML. Lipid recovery from wet oleaginous microbial biomass for biofuel production: A critical review. Appl Energy 2016;177:879–95. https://doi.org/10.1016/j.apenergy.2016.06.002.

[4] Gill DS, Rana D. Preparation of Some Novel Copper(I) Complexes and their Molar Conductances in Organic Solvents. Z Für Naturforschung A 2009;64:269–72. https://doi.org/10.1515/zna-2009-3-416.
